# The F1F3 Recombinant Chimera of *Leishmania donovani*-Nucleoside Hydrolase (NH36) and Its Epitopes Induce Cross-Protection Against *Leishmania (V.) braziliensis* Infection in Mice

**DOI:** 10.3389/fimmu.2019.00724

**Published:** 2019-04-09

**Authors:** Marcus Vinícius Alves-Silva, Dirlei Nico, Paula Melo de Luca, Clarisa B. Palatnik de-Sousa

**Affiliations:** ^1^Laboratório de Biologia e Bioquímica de Leishmania, Departamento de Microbiologia Geral, Instituto de Microbiologia Paulo de Góes, Universidade Federal do Rio de Janeiro, Rio de Janeiro, Brazil; ^2^Programa de Pós-Graduação em Biotecnologia Vegetal e Bioprocessos, Centro de Ciências da Saúde, Universidade Federal do Rio de Janeiro, Rio de Janeiro, Brazil; ^3^Laboratório de Imunoparasitologia, Instituto Oswaldo Cruz, Rio de Janeiro, Brazil; ^4^Faculdade de Medicina, Instituto de Investigação em Imunologia, Universidade de São Paulo, São Paulo, Brazil

**Keywords:** *Leishmania* (*V*.) *braziliensis*, cutaneous leishmaniasis, mucocutaneous leishmaniasis, nucleoside hydrolase NH36, F1F3 recombinant chimera, mixed or T-cell regulatory response

## Abstract

*Leishmania* (*V*.) *braziliensis* is the etiological agent of Cutaneous (CL) and Mucocutaneous leishmaniasis (ML) in the New World. CL can be more benign but ML can be severe and disfiguring. Immunity to these diseases include hypersensitivity, an enhanced inflammatory response with strong IFN-γ and TNF-α secretion. Additionally, the production of IL-10 which down modulates the immune response is reduced. The Nucleoside hydrolase (NH36) of *Leishmania* (*L*.) *donovani* is the main antigen of the Leishmune veterinary vaccine and its F3 domain induces a CD4^+^ T cell-mediated protection against *L*. (*L*.) *infantum chagasi* infection. Prevention of *L*. (*L*.) *amazonensis* infection requires in contrast an additional CD8^+^ T cell mediated response induced by the F1 domain. Consequently, the F1F3 recombinant chimera, which contains both domains cloned in tandem, optimized the vaccine efficacy against *L*. (*L*.) *amazonensis* mouse infection. We compared the efficacies of NH36, F1, F3, and the FIF3 chimera against *L*. (*V*.) *braziliensis* mouse infection. The F1F3 chimera increased the NH36 specific IgA and response before and after infection and the IgG and IgG3 levels after challenge. It also induced a 49% stronger intradermal response to leishmanial antigen (IDR) than NH36 that was positively correlated to the levels of IFN-γ and TNF-α, IgG, IgG2a, IgG2b, and IgG3 anti-NH36 antibodies. However, stronger Th1 responses with elevated IFN-γ*/*IL-10 and TNF-α*/*IL-10 ratios were promoted by the F3 and F1 vaccines and detected in infected controls while the F1F3 chimera promoted the highest IL-10 secretion, which reduced the pathological Th1 response, and characterized the induction of a mixed and/or T-cell regulatory response. We identified the epitopes responsible for these immune responses. The F3 vaccine induced the earliest immunity and after challenge, the F1F3 chimera promoted the highest CD4^+^ and CD8^+^ cytokine-secreting T cell responses, and the predominant frequencies of multifunctional CD4^+^ and CD8^+^IL-2^+^TNF-α^+^IFN-γ^+^ T cells. Also as observed against *L*. (*L*.) *amazonensis* infection, the F1F3 chimera showed the strongest reduction of the ear lesions sizes induced by *L*. (*V*.) *braziliensis*. Our results confirm the potential use of the F1F3 chimera in a multi-species cross-protective vaccine against *L*. (*V*.) *braziliensis*.

## Introduction

Leishmaniasis comprises a complex of diseases which are endemic in 98 countries and about 350 million individuals are at risk. The different forms of the disease include: Visceral leishmaniasis (VL), Cutaneous leishmaniasis (CL), Mucosal leishmaniasis (ML), Diffuse cutaneous leishmaniasis (DCL), and Disseminated leishmaniasis (DL) (1). While VL and DCL represent the most severe forms of the disease and show a characteristic impaired cellular immune response, CL and ML present a Th1 immune response with high secretion of the pro-inflammatory cytokines IFN-γ and TNF-α (2, 3).

In Brazil the main species that causes CL and ML is *Leishmania* (*V*.) *braziliensis*. CL is considered the most common manifestation of tegumentary leishmaniasis and shows a primary lesion that develops at the site of the bite of the transmitting insect (4–6). Lesions, which appear as ulcers with elevated margins, are usually found in uncovered regions of the body, such as the face, arms, legs, and evolve over weeks or months (7). Lymphadenopathy is also detected in CL at early stages of the infection (8). ML, on the other hand, is characterized by single or multiple lesions (4) which in 90% of the cases are located in the nasal mucosa, although the lips, palate, mouth, pharynx and larynx, as well as the ears, can also be affected (9). ML occurs in 3% of the patients that had previously developed CL, damaging tissues and often with disfiguring lesions on the face (8) which may cause severe psychological problems to such patients. Recent investigations have disclosed that the severity of CL caused by *L*. (*V*.) *braziliensis* is more related to an exacerbated inflammatory response, than to a high parasite burden (8, 10–13).

Although the secretion of IFN-γ by macrophages has been associated with the killing of intracellular *Leishmania*, recent studies have revealed the role of IFN-γ and TNF-α in the pathology of CL and ML lesions (12). There is some evidence obtained in patients with CL that supports this notion: (1) a higher secretion of the pro-inflammatory cytokines IFN-γ and TNF-α by lymphocytes (14, 15), (2) the presence of rich inflammatory infiltrates in lesions where parasites are not usually found, (3) a good correlation between the frequency of inflammatory cytokine producing T cells and lesion size (8), and (4), the findings of high frequencies of cells expressing IFN-γ and low frequencies of cells expressing the IL-10 receptor (16) or IL-10 (6, 17, 18). IL-10 regulates the expression and the effector and secretory functions of IFN-γ that determine the course of the *Leishmania* infection (19). Additionally, the presence of IFN-γ-secreting CD8^+^ T cells was associated with the Th1 response against the Cutaneous infection by *L*. (*L*.) *major* in Iran (20).

The present drug therapy, which is highly toxic to patients, has not been effective in eradicating CL, and furthermore, the parasites have exhibited an increased resistance to these drugs worldwide (21–23). Vaccines on the other hand, could be important weapons for control and prevention (24). However, no effective vaccine against the human form of the infection by *L*. (*L*.) *braziliensis* or any other *Leishmania* spp currently exists (25).

The first generation of vaccines against leishmaniasis was developed mainly against CL and was obtained by manipulating dead parasites with or without adjuvants (26). The second generation of vaccines can be divided into three categories according to their composition: (1) live vaccines containing genetically modified *Leishmania* or viruses expressing *Leishmania* genes (2) defined, recombinant or synthetic vaccine fractions or subunits, and (3) vaccines with partially purified native fractions Third generation vaccines contain cloned antigen genes in eukaryotic promoter vectors injected and translated directly into the muscle (27–29).

Our laboratory developed the first licensed vaccine against canine visceral leishmaniasis, called Leishmune®, which is composed of the FML complex antigen of *Leishmania* (*L*.) *donovani* (28, 30). The use of Leishmune® in Brazilian endemic areas successfully reduced the canine and human incidence of the disease (31). The main antigen of the FML complex is the Nucleoside hydrolase NH36 of *Leishmania* (*L*.) *donovani*, which proved itself to be a strong immunogen in both, its recombinant protein and DNA forms (32, 33). NH36 is a strong phylogenetic marker present in all the leishmania species studied until now, and a high homology has been found between the amino acid sequences of NH36 of *Leishmania* (*L*.) *donovani* and the NHs of *L*. (*L*.) *major* (95%) (34), *L*. (*L*.) *infantum chagasi* (99%), *L*. (*L*.) *infantum* (99%), *L*. (*L*.) *amazonensis* (93%) (35), *L*. (*L*.) *mexicana* (93%), *L*. (*L*.) *tropica* (97%), and *L*. (*V*.) *braziliensis* (84%) (36).

Accordingly, vaccination with the NH36 protein formulated with saponin induced cross-protection. It prevented and cured mice from infections caused by *L*. (*L*.) *infantum chagasi* (33), *L*. (*L*.) *amazonensis* (35, 37), and *L*. (*L*.) *mexicana* (33). The immunity against NH36 in vaccinated mice with visceral leishmaniasis (VL) is mediated by a Th1 CD4^+^ T cell response against its C-terminal moiety, the F3 domain (38), and is correlated to a strong secretion of TNF-α and an enhanced intradermal response to the leishmanial antigen. On the other hand, besides the CD4^+^ T cell response against the F3 domain, the vaccine immune response generated against CL caused by *L*. (*L*.) *amazonensis* is also mediated by an additional CD8^+^ T cell response directed against the N-terminal moiety of NH36, called the F1 domain (35, 39). In order to increase the vaccine efficacy against CL we recently cloned, the F1 and F3 domains in tandem, and observed that vaccination against *L*. (*L*.) *amazonensis* infection with the F1F3 recombinant chimera determined the largest reductions in the sizes of lesions and parasite loads, and enhanced the antibody responses. Also this vaccine with the F1 and F3 domains in tandem enhanced the intradermal response to leishmanial antigen (IDR), the IFN-γ and IL-10 secretion and the proportions of CD4^+^ multifunctional T cells secreting IL-2, TNF-α, and IFN-γ (39), which have been pointed out as having the best effector and memory T-cell functions (40).

Since *L*. (*V*.) *braziliensis* is the main agent of CL and ML in Brazil and Latin America and its NH exhibits high homology to the sequence of NH36, we decided to investigate the immunogenic potential and vaccine efficacy induced by the F1F3 chimera or the F1 and F3 domains, against infection in mice by *L*. (*V*.) *braziliensis*. The goal of this study is to contribute to the development of a universal cross-protective vaccine against leishmaniasis.

## Materials and Methods

### Recombinant Antigen Expression and Purification

The Nucleoside Hydrolase NH36 is a 314 amino acids protein (Genbank access number AY007193, SwissProt-UniProt access number Q8WQX2-LEIDO). The expression vector pET-28b was cloned with either the N-terminal (F1, amino acid sequences 1-103), the central (F2, amino acids 104-198), or the C-terminal (F3, amino acids 199-314) domains of NH36, using a 6 His-Tag at their C-terminals, between the restriction sites of *Nco*I and *Xho*I (35, 37, 39). Additionally, the F1F3 recombinant chimera which includes the F1 and the F3 protein cloned in tandem, was obtained with optimized codons (Genscript, NJ, USA).

For each expression experiment, *E. coli* BL21DE3 bacteria were transformed with plasmids pET28bF1, pET28bF3, or pET28bF1F3 (GenScript, NJ, USA) and stored at −80°C. After expression induction, the bacterial pellet maintained on ice was resuspended in 20 ml of sonication buffer (57.5 mM NaH2 PO4, 128.7 mM NaCl, 500 mL distilled water, pH 7.0) sonicated for 1 min and 30 s with cycles of 2 and 10 s intervals (Fisher Scientific 500 Sonic Dismembrator). Thereafter, the sonicated material was centrifuged at 14,000 rpm at 4°C for 20 min. The culture supernatants, after expression, were discarded since the F1, F3, and F1F3 chimera proteins were present in greater amounts in the pellets. Purification of the proteins was performed by Nickel column affinity chromatography according to the Ni-NTA resin manufacturer's instructions (Qiagen). The recombinant antigens were recovered in 10 volumes of urea buffer pH 4.5, dialyzed overnight against PBS at 4°C and preserved in PBS with 1 mM PMSF at −80°C (38). The absence of LPS was confirmed using the LAL QCL-1000 kit (Lonza).

### Immunization and Challenge

Groups of 2-month-old BALB/c females were randomized by body weight and immunized with three subcutaneous doses of 100 μg of NH36, F1, F3, or F1F3 chimera formulated with 100 μg of Riedel de Haen saponin (Sigma, St. Louis, MO, USA) in 0.2 ml 0.9% NaCl salt solution, in the back at weekly intervals. There were two other groups: the control group, which received no immunization and the saline group. One week after the complete immunization the animals were infected by the intradermal route in the ears with 5 x 10^6^ stationary phase promastigotes *L*. (*V*.) *braziliensis* (MCAN/BR/98/R69) obtained after 3 passages in culture. Briefly, to obtain infective promastigotes of *L*. (*V*.) *braziliensis*, the samples were kept cryopreserved in liquid nitrogen and at the time of use were thawed at room temperature in Schneider's medium with 20% of SBF, L glutamine, Kanamycin (30 μg/ml), and 2% filtered human urine obtained from a male subject.

### Measurement of Serum Antibody Response

The NH36 specific antibody response was evaluated to detect IgA, IgM, IgG1, IgG2a, IgG2b, and IgG3 immunoglobulins from animal sera 1 week after the last immunization and after the eighth week of infection. Greiner plates (96 wells) were sensitized with 50 μl of recombinant NH36 protein (40 μg/ml) in carbonate/bicarbonate buffer pH 9.6; and incubated for 1 h at 37°C and overnight at 4°C. Thereafter, the plates were subjected to five washes with PBS^**^ (0.018 M PBS pH 7.2, 1% skimmed milk and 0.05% Tween 20) and subsequent incubation with the dilutions of the sera also in PBS ^**^, for 1 h at 37°C. The plates were washed again five times with PBS ^**^, and 50 μl of goat anti-IgA, IgM, IgG1, IgG2a, IgG2b, and mouse IgG3 antibodies conjugated with peroxidase (Southern Biotechnology Associates, Birmingham, AL, USA) or 50 μl of peroxidase-protein-A conjugate (Kirkegaard & Perry Laboratories, Gaithersburg, Maryland, USA) were added at a 1: 1,000 dilution in PBS ^**^. Plates were further incubated for 1 h and washed five times with PBS ^**^ and the reaction was developed with 50 μl/well of OPD solution (Orto Phenylene Diamine–Sigma) in OPD buffer pH 5.2, for 30 min, in the dark. The reaction was interrupted with 10 μl/well of 1 N sulfuric acid and the plates were read in a Benchmark BIO-RAD microplate reader with a 492 ηm filter. Triplicates of each serum were used, at 1/100 dilution in double-blind tests.

### Intradermal Skin Test (IDR)

To evaluate the IDR response, the animals were inoculated with 100 μl of the lysate of 10^7^ stationary phase *L*. (*V*.) *braziliensis* promastigotes in the right hind paw pads, while 100 μl of saline was injected into the left hind paws, as a negative control. To prepare the parasite lysate, promastigotes of *L. (V.) braziliensis* were cultured in Schneider medium supplemented with 10% Fetal calf Serum and 2% human urine. After 5 days of *in vitro* culture, the log-phase promastigotes were transferred to a 1 liter Erlenmeyer containing 200 ml of culture medium and incubated at 28°C for 3 days until reaching the stationary stage. Cells were then centrifuged at 4°C 6,000 g, washed (3 times) in 0.9% NaCl solution and counted in a Neubauer's chamber. A suspension of 10^8^ promastigotes/ml in saline solution was produced and subjected to freezing in a liquid Nitrogen bath and thawing under a stream of water alternately, for five consecutive times to obtain the lysate. The measurements of the paw thicknesses (mean of 5 measurements/animal) were performed with a Mitutoyo® caliper at 0, 24, and 48 h after inoculation of the lysate. The IDR response was expressed as the difference between the paw thickness before and after lysate inoculation. For each measurement the value of its respective saline contralateral control paw was subtracted (39).

### Secreted Cytokines-Assay

Spleens of NH36, F1, F3, and F1F3 vaccinated and control mice, were removed aseptically after euthanasia, before and after infection. Splenocyte suspensions were obtained in RPMI medium (Sigma-Co) supplemented with 10% fetal bovine serum, 1% L-glutamine and 5 mM 2-β-mercaptoethanol. Cells were further counted in a hemocytometer, plated into Costar 96-well plates (10^6^*/*well) and incubated with or without 25 μg/ml of recombinant NH36, for 5 days with 5% CO_2_ at 37°C, according to prior standardization (39, 41). The levels of IFN-γ, TNF-α, and IL-10 were then assayed in supernatants diluted in the blocking solution, using the specific BD OptEIA Mouse kits ELISA Set II (BD Biosciences), following the manufacturer's instructions. The absorbance reading was performed using a BIORAD Benchmark Microplate Reader apparatus with a 570 ηm filter.

### Intracellular Cytokine Staining (ICS) and Flow Cytometry

Aliquots of 10^6^ spleen cells of NH36, F1, F3, and F1F3 vaccinated and control mice, before and after infection were diluted in supplemented RPMI medium and plated into Costar 96 well-plates. Cultures were stimulated with 25 μg/ml of recombinant NH36 or with 25 μg/ml of the synthetic peptides and the wells without the stimulus were used as negative control. The cells were incubated in a 5% CO_2_ oven at 37°C for 24 h according to prior standardization. The intracellular production of IL-2, TNF-α, and IFN-γ by CD4^+^ and CD8^+^ lymphocytes was determined by multi-parametric analysis after incubation with brefeldin (SIGMA) at a final concentration of 10 μg/ml for 4 h at 37°C under 5% CO_2_, prior to intracellular labeling. After washing with FACS buffer (2% fetal bovine serum, 0.1% sodium azide in PBS), the splenocytes were labeled for 20 min at 4°C in the dark, with anti-mouse monoclonal antibodies CD4FITC (clone GK1.5) and CD8FITC (clone 53-6.7) (R & D systems, Inc) in FACS buffer (1/100) and fixed with 4% paraformaldehyde. Additionally, the cells were washed and treated with FACS buffer containing 0.5% saponin (SIGMA) for 20 min at room temperature and further stained with IFN-γAPC, IL-2-PerCP-Cy5.5, and TNF-αPE (BD) monoclonal antibodies Pharming), diluted 1/100 in FACS buffer containing 0.5% saponin for 20 min, and finally washed and suspended in FACS buffer. For control, 10^6^ splenocytes from each animal tested were also incubated without the addition of antigen. A total of 100,000 cells were analyzed in a Becton Dickinson FACScalibur apparatus. The data was analyzed by the Flow-jo program (Treestar, USA) using Bolean gates combinatory analysis. Briefly the gated single-cell lymphocyte population was additionally gated for CD4 and CD8 expression. Production of each cytokine (IL-2, TNF-α, and IFN-γ) were analyzed individually within CD4 + or CD8 + lymphocyte gates. Boolean gating was used to generate combinations of cytokine expression in order to identify lymphocytes expressing only one cytokine or any combination of two or three cytokines simultaneously.

### Epitope Assays

Splenocytes from mice vaccinated with the F1F3 chimera or treated with saline solution and subsequently challenged with 5 x 10^6^ promastigotes of *L*. (*V*.) *braziliensis* were cultured, in the eighth week of infection, *in vitro* with 25 μg/ml of NH36, or with each of the predicted CD4 epitopes for the F1 domain (ELLAITTVVGNQ and DVAGIVGVPVAAGCT) and F3 domain (FMLQILDFYTKVYE, FRYPRPKHCCHTQVA, and KFWCLVIDALKRIG), with the highly scored epitope for CD8^+^ T cells of the F1 domain (YPPEKTKL) or with the mixture of all epitopes,. Cells from F3 vaccinated mice were incubated with the epitopes for CD4^+^ T cells of F3 only (38, 39). The supernatants were assayed for secretion of IFN-γ, TNF-α, and IL-10, and the lymphocytes were assayed for expression of IL-2, IFN-γ, TNF-α using the methodology described above.

### Evolution of Infection and Quantification of Parasite Load

The evolution of lesions in the infected ears was evaluated weekly with a Mitutoyo® caliper and compared by subtraction of the values of the contra-lateral uninfected ears. At the end of week 8, mice were ethically euthanized by intraperitoneal injection of Ketamine (250 mg/kg) and Xylazin (50 mg/Kg) to induce anesthesia. The euthanasia was further confirmed in a CO_2_ chamber.

In addition, the parasite load in lesions was evaluated by a Limiting Dilution Assay (LDA). The infected ears were removed aseptically after euthanasia, immersed in 70% ethanol for 10–15 min and further dried in the air on sterile filter paper. With the aid of tweezers, the dorsal part of the ears was separated from the ventral part, exposing the dermis which was washed with Schneider's medium to eliminate the skin. The remainder of the ear was minced into small pieces and added to 1 ml of Schneider's supplemented medium. This 1 ml suspension was added to the first well of a 24-well culture plate and diluted 1/5 times, followed by incubation at 26°C for 4 days, with daily observation under the inverted microscope (39). The titer was given to the last dilution containing *Leishmania* where the total number of promastigotes were also quantified with a hemocytometer.

### Statistical Methods

Differences between variables were analyzed by the non-parametric tests of Kruskall Wallis and Mann Whitney (GraphPad Prism6 program). The IC95% test was used for comparison of the parasite loads by LDA. Correlations analyses were performed using the bivariate Pearson test (GraphPad Prism6 program). All experiments were performed at least twice, and the error bars are SE based.

## Results

### The F1F3 Chimera Enhances the Antibody Response

**Figure 1:**
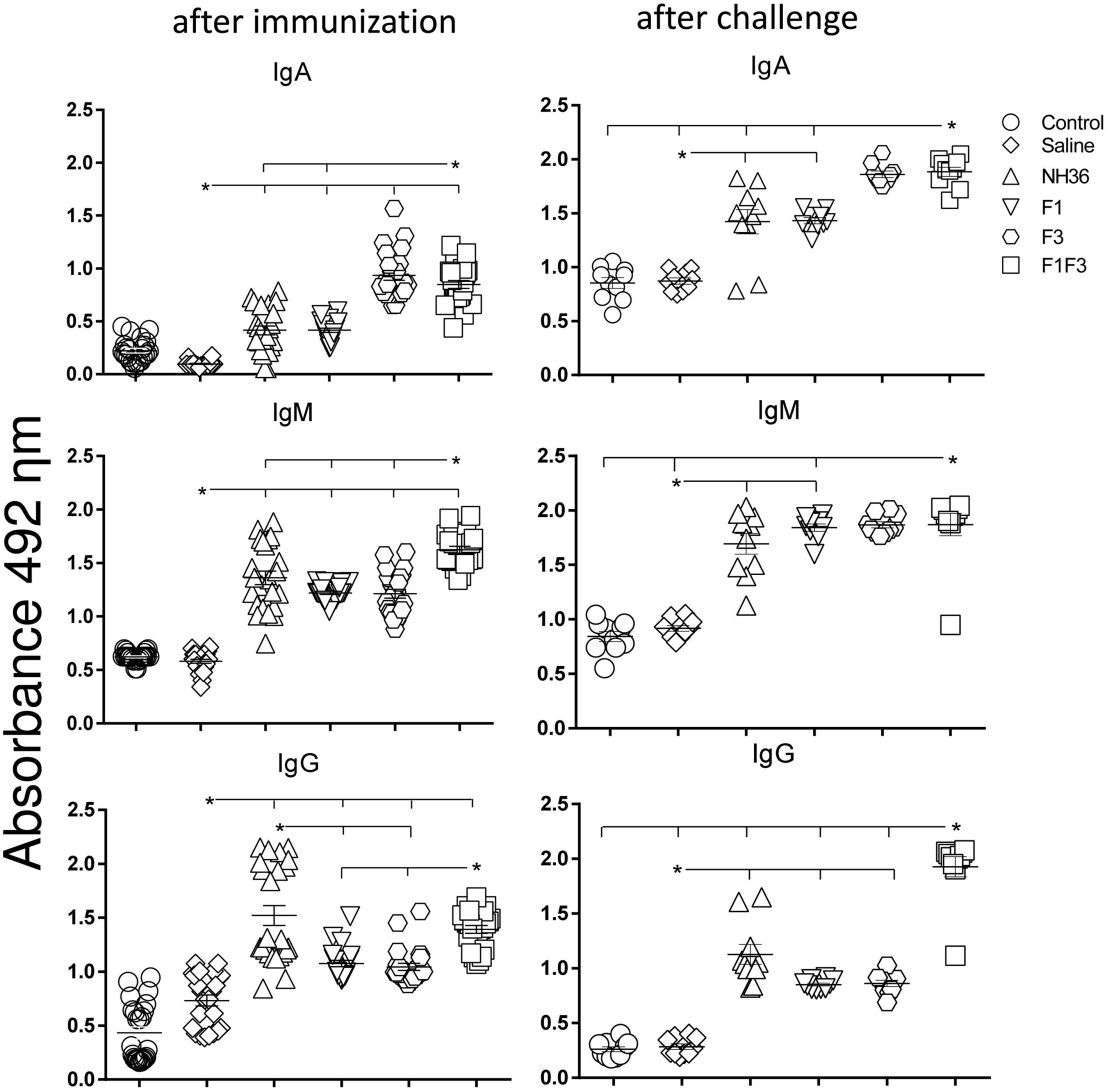
Development of NH36 specific IgA, IgM and IgG antibodies after prophylactic vaccination. The results represent the individual NH36 specific antibody absorbance data in 1/100 diluted sera measured by ELISA assay in the sera of mice after prophylactic immunization and at the end of week 8 after infection with *L*. (*V*.) *braziliensis*. Statistical differences were assessed using the Kruskal Wallis and Mann Whitney methods. The horizontal bars represent the means of two independent experiments with *n* = 10 mice per group, after immunization. Asterisks and horizontal lines indicate significant differences between treatments (*p* < 0.001).

**Figure 2:**
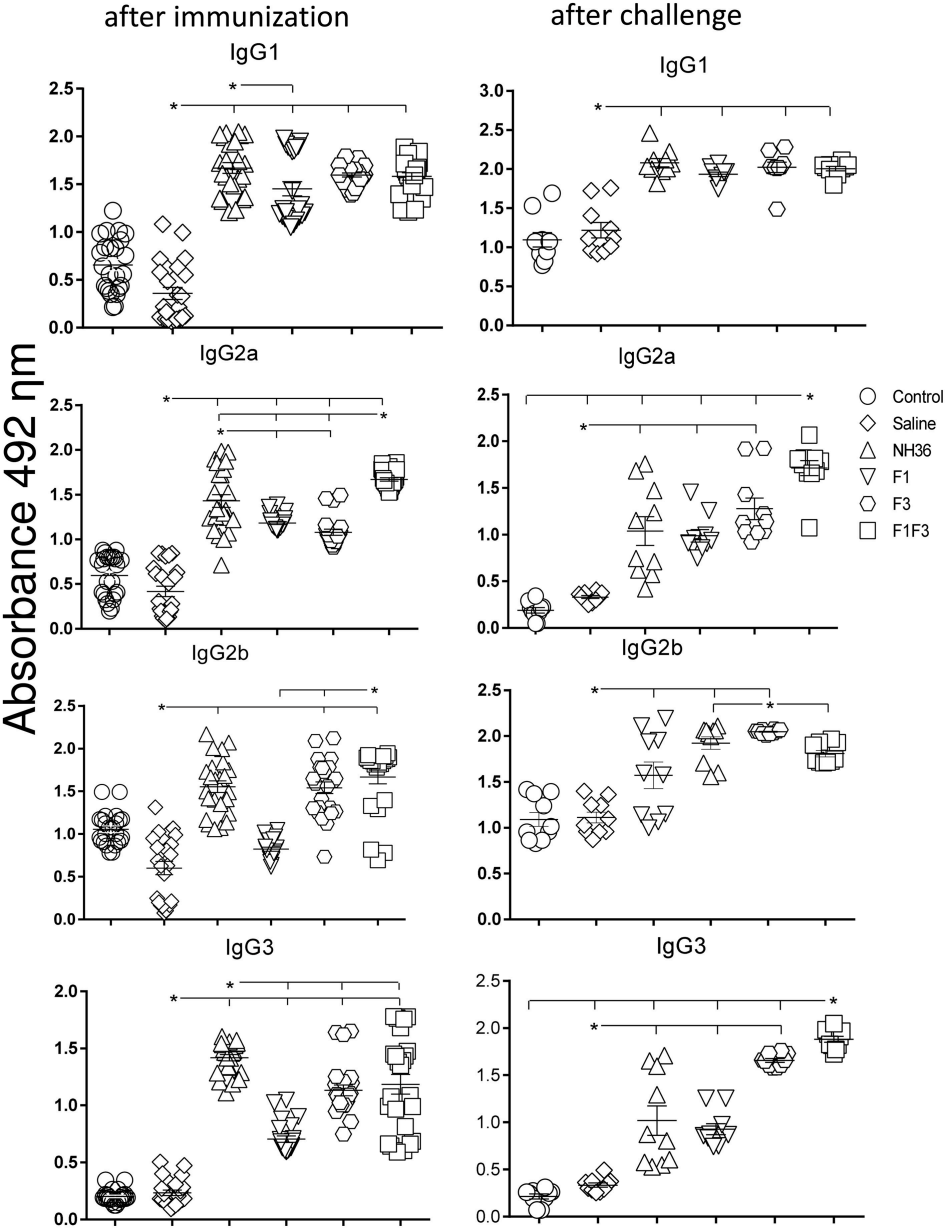
Development of NH36 specific IgG1, IgG2a, IgG2b, IgG3 subtype antibodies. The results represent the individual NH36 specific antibody absorbance data in 1/100 diluted sera measured by ELISA assay in the sera of mice after prophylactic immunization and at the end of week 8 after infection with *L*. (*V*.) *braziliensis*. The chimera vaccine induced the highest Ig2a, Ig2b after immunization. After challenge chimera induced the highest Ig2a and IgG3. Statistical differences were assessed using the Kruskal Wallis and Mann Whitney methods. The horizontal bars represent the means of two independent experiments with *n* = 10 mice per group. Asterisks ^*^ and horizontal lines indicate significant differences between treatments (*p* < 0.001).

We investigated the protective potential of three *sc* doses of the NH36, F1, F3, or F1F3 recombinant vaccines formulated with saponin, to prevent the development of *L*. (*V*.) *braziliensis* infection in the ears of BALB/c mice. The F1F3 chimera increased the NH36 specific IgA (Figure 1) and IgG2a (Figure 2) antibody responses, and was stronger than the other formulations, both before and after infection, while it enhanced the IgM and titers before infection, and the IgG (Figure 1) and IgG3 levels after challenge (Figure 2).

Additionally, the chimera was as potent as the NH36 protein, in generation of IgG antibodies before infection, and in the IgG1 and IgG2b response (Figure 2), before and after challenge.

We identified significant associations between the IgG, IgG2a, IgG2b, and IgG1 antibody absorbencies, both before and after infection (*p* = 0.0009 for all comparisons). After challenge for instance, the increases in IgG and IgG2a (*p* < 0.0001, *R* = 0.8652, *R*^2^ = 0.7487), IgG2b (*p* < 0.0001, *R* = 0.5532, *R*^2^ = 0.3061) and IgG1 (*p* < 0.0001, *R* = 0.6827, *R*^2^ = 0.4461) were highly correlated.

### The F1F3 Chimera Increases the Intradermal Response to *L*. (*V*.) *braziliensis* Antigen

**Figure 3:**
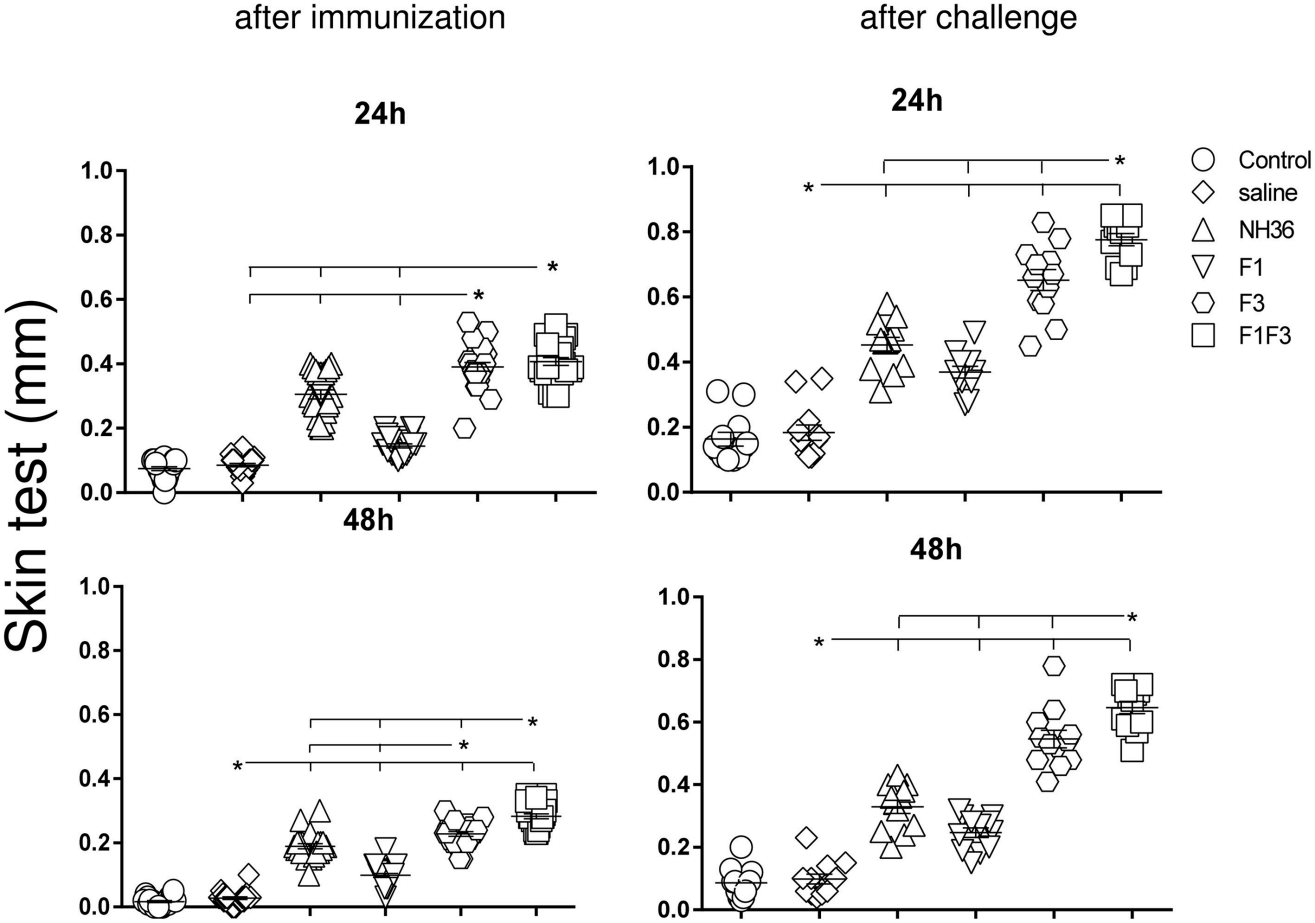
Intradermal response against *L*. (*V*.) *braziliensis*. IDR was performed with lysate of *L*. (*V*.) *braziliensis* after immunization and after challenge, at 24 and 48 h after antigen injection. The bars represent the means of each experimental group of two independent experiments with *n* = 10 mice per group after immunization, and *n* = 7 mice per group, after challenge. Statistical differences were assessed using the Kruskal Wallis and Mann Whitney methods. Asterisks ^*^ and horizontal lines indicate significant differences between treatments (*p* < 0.0001).

The IDR response to the lysate of *L*. (*V*.) *braziliensis* promastigote was assayed 24 and 48 h after the antigen injection as well as after complete immunization and after challenge (Figure 3). The strongest IDR responses were observed in animals vaccinated with the F1F3 chimera for all the times evaluated. Confirming its superiority, in fact, the chimera induced 25 and 33% significantly stronger responses than the NH36 vaccine (*p* < 0.0001), at 24 and 48 h after immunization (Figure 3). After challenge, the IDRs were even enhanced, and the chimera vaccine induced 42 and 49% stronger responses than the NH36 vaccine, at 24 and 48 h after injection, respectively (Figure 3). Noteworthy, the F3 vaccine was as potent as the chimera, before infection at 24 h, but the chimera recovered its superiority at 48 h. As occurred for most antibodies, the F1 vaccine also induced, the lowest IDR responses (Figures 1–3).

After immunization, the IDR responses were positively correlated with the increases of IgA, IgM, IgG and all subtypes of IgG antibodies, at 24 and 48 h (*p* < 0.05, for all comparisons). After infection, the correlation indexes were even stronger. For instance, at 48 h after antigen injection, the IDR was positively correlated to the IgA (*p* < 0.0001, *R* = 0.8056, *R*^2^ = 0.6490), IgM (*p* < 0.0001, *R* = 0.6753, *R*^2^ = 0.4560), IgG (*p* < 0.0001, *R* = 0.7398, *R*^2^ = 0.5473), IgG1 (*p* < 0.0001, *R* = 0.6662, *R*^2^ = 0.4438), IgG2a (*p* < 0.0001, *R* = 0.8340, *R*^2^ = 0.6956), IgG2b (*p* < 0.0001, *R* = 0.6082, *R*^2^ = 0.3700), and IgG3 (*p* < 0.0001, *R* = 0.8610, *R*^2^ = 0.7412) antibody responses.

### F1F3 Chimera and F3 Vaccine Candidate Promote the Secretion of IFN-γ, TNF-α, and IL-10

**Figure 4:**
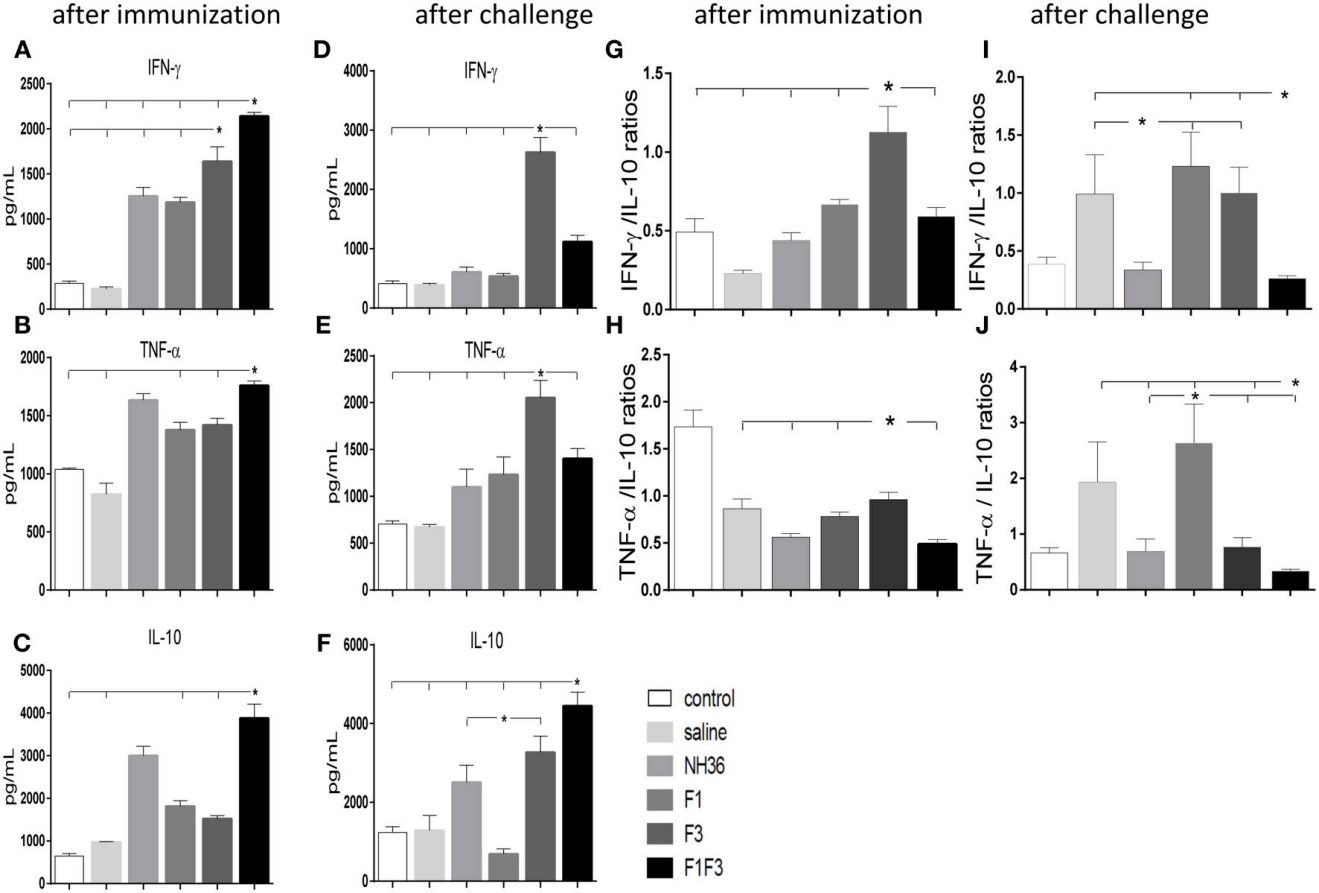
Th1 NH36 specific cytokine responses in supernatants of splenocyte cultures after immunization and after challenge. Secretion of IFN-γ **(A,D)**, TNF-α **(B,E)** and IL-10 **(C,F)** were evaluated after immunization and after challenge, respectively, in the supernatants of splenocytes cultured for 3 days *in vitro* with the recombinant NH36 antigen using an ELISA assay with recombinant IFN-γ, TNF-α and IL-10 as standards. The Th1 responses are expressed as the calculation of the secreted IFN-γ*/*IL-10 **(G,I)** and TNF-α*/*IL-10 **(H,J)** ratios, after immunization and after challenge, respectively. Individual and mean cytokine concentrations are represented in pg/ml corresponding to two independent experiments with *n* = 6 animals after immunization, and 7–8 animals after challenge for each treatment. Statistical differences were assessed using the Kruskal Wallis and Mann Whitney methods. Asterisks ^*^ and horizontal lines indicate significant differences between treatments (*p* < 0.05).

The NH36 specific splenocyte cytokine secretions were evaluated in the supernatants (Figures 4A–F). Before infection, the F1F3 chimera enhanced the IFN-γ secretion above the levels promoted by all other vaccines (Figure 4A). Second to F1F3, the F3 domain was also more potent that the NH36 and F1 vaccines. Additionally, after immunization the chimera shared its superior secretion of TNF-α and IL-10 with the NH36 vaccine. In contrast, after infection, the F3 vaccine induced the strongest IFN-γ (Figure 4D) and TNF-α secretion (Figure 4E), while the chimera, followed by the F3 and NH36 vaccines, was still predominant for the production of IL-10 (Figures 4C,F).

After immunization, the IFN-γ secretion was positively correlated to the IgG2a (*p* = 0.0322, *R* = 0.2769, *R*^2^ = 0.0768) and IgG3 (*p* < 0.0001, *R* = 0.8025, *R*^2^ = 0.6439) antibodies and to the IDR, at 24 h (*p* < 0.0001, *R* = 0.7677, *R*^2^ = 0.5894) and 48 h (*p* < 0.0001, *R* = 0.8417, *R*^2^ = 0.7085). The TNF-α secretion was also correlated to the IgG1 (*p* = 0.0380, *R* = 0.2686, *R*^2^ = 0.0721), IgG2a (*p* < 0.0001, *R* = 0.5116, *R*^2^ = 0.2618), IgG2b (*p* = 0.0006, *R* = 0.4301, *R*^2^ = 0.1856) and IgG3 (*p* < 0.0001, *R* = 0.6940, *R*^2^ = 0.4817) antibodies and to the IDR, at 24 h (*p* < 0.0001, *R* = 0.6940, *R*^2^ = 0.4678) and 48 h (*p* < 0.0001, *R* = 0.7779, *R*^2^ = 0.6052). Additionally, secretion of IL-10 was also correlated the IgG2a (*p* = 0.0002, *R* = 0.4592, *R*^2^ = 0.2109), IgG2b (*p* = 0.0120, *R* = 0.3223, *R*^2^ = 0.1039) and IgG3 (*p* < 0.0001, *R* = 0.5660, *R*^2^ = 0.3203) antibodies and to the IDR, at 24 h (*p* < 0.0001, *R* = 0.6665, *R*^2^ = 0.4442), and 48 h (*p* < 0.0001, *R* = 0.7408, *R*^2^ = 0.5488). These results suggested the presence of a potential mixture of a T regulatory response induced by the chimera and a Th1 response induced by the F3 vaccine.

Calculation of the IFN-γ*/*IL-10 and TNF-α*/*IL10 ratios confirmed this mixed response hypothesis (Figures 4G–J). We found that after immunization, the highest IFN-γ*/*IL-10 ratios, which indicate the highest Th1 response, were induced by the F3 vaccine, while the NH36, F1, and F1F3 chimera vaccines determined ratios below 1 (Figure 4G). These low ratios indicate that secretion of IL-10 was higher than that of the pro-inflammatory cytokines in the vaccines that contained the F1 domain. Additionally, a similar Th1 profile with elevated secretion of TNF-α/IL10 ratios was also revealed in the *L* (*V*.) *braziliensis* infected mice (saline group) and in the F3-vaccinated mice, but not in the NH36 or F1F3 chimera vaccinated animals (Figure 4H). Remarkably, at the end of week 8 after infection, Th1 responses were observed in the saline treated, as well as in the F1 and F3-vaccinated mice that exhibited increased IFN-γ*/*IL-10 ratios (Figure 4I), and in the saline treated and F1-vaccinated mice, that showed elevated TNF-α*/*IL10 ratios (Figure 4J). In contrast, the F1F3 chimera followed by the NH36 vaccine showed the lowest ratios which are indicative of the strongest regulatory response (Figures 4I,J).

**Figure 5:**
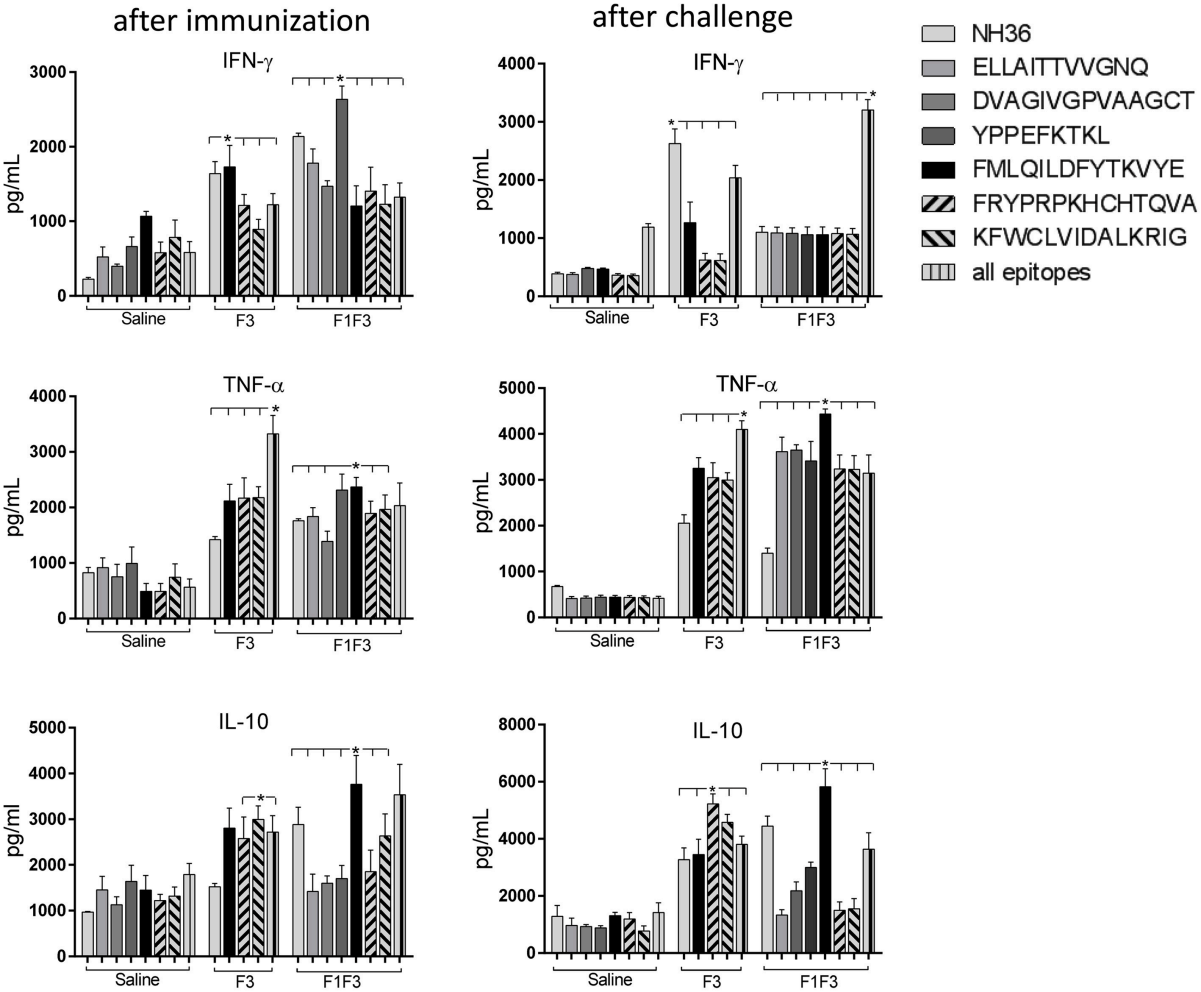
Cytokine assay in supernatants of splenocytes stimulated with the NH36 epitopes. Splenocytes of mice vaccinated with 100 μg of the F3 domain were incubated *in vitro* with 25 μg*/*ml of NH36, and with each of the predicted CD4^+^ epitopes for the F3 domain (FMLQILDFYTKVYE, FRYPRPKHCCHTQVA, and KFWCLVIDALKRIG) or with the mixture of all the epitopes, after immunization and at the end of week 8 after infection. In addition, splenocytes of the control infected mice and of mice vaccinated with 100 μg of the F1F3 chimera were incubated *in vitro* with 25 μg*/*ml of NH36, with each one of the predicted CD4^+^ epitopes for the F1 domain (ELLAITTVVGNQ and DVAGIVGVPVAAGCT) and F3 domain (FMLQILDFYTKVYE, FRYPRPKHCCHTQVA, and KFWCLVIDALKRIG), and with the highly scored epitope for CD8^+^ T cells of the F1 domain (YPPEKTKL), after immunization and after infection. Secretions of IFN-γ, TNF-α, and IL-10 were measured by an ELISA assay in the supernatants of splenocytes and expressed in pg/ml. Data are means + SE of two independent experiments, each one with 8–10 animals per treatment. Statistical differences were assessed using the Kruskal Wallis and Mann Whitney methods. Asterisks ^*^ and horizontal lines indicate significant differences between treatments (*p* < 0.0001).

The F1F3 chimera and the F3 vaccines induced the strongest secretion of IFN-γ, TNF-α and IL-10. So we decided to identify, which epitopes of the F3 and the F1 domains these responses were directed at. Therefore, splenocytes of mice vaccinated with F3 were incubated with NH36 and the three MHC Class II-restricted synthetic epitopes located in F3, while cells of saline-treated and F1F3 vaccinated mice were stimulated with NH36 and with both, the MHC Class I and II restricted epitopes of F1, and the MHC Class II restricted epitopes of F3 (39) (Figure 5). The identity of the *L. (L.) donovani* NH36 and the *L. (V.) braziliensis* NH sequences was analyzed using the Blast-Pubmed tool (Supplementary Figure 1). The ELLAITTVVGNQ epitope is identical in both NHs while the YPPEFKTKL differs in one, the FRYPRPKHCTQVA and DVAGIVGVPVAAGCT in two, and the FMLQILDFYTKVYE and KFWCLVIDALKRIG in three amino acids, respectively.

Indicating their immunogenic predominance the epitopes were more potent than NH36, in secretion of TNF-α and IL-10 (Figure 5). After immunization, cells of mice vaccinated with the chimera showed the strongest IFN-γ and TNF-α responses against the YPPEFKTKL epitope of F1 (Figure 5) and the strongest TNF-α and IL-10 response against the FMLQILDFYTKVYE epitope of F3. After infection, the maximal TNF-α and IL-10 response generated by the chimera was also directed against the FMLQILDFYTKVYE epitope while all other epitopes only induced TNF-α secretion. The F3 vaccine, on the other hand, promoted similar TNF-α and IL-10 secretion against the FRYPRPKHCCHTQVA and KFWCLVIDALKRIG epitopes (Figure 5).

**Figure 6:**
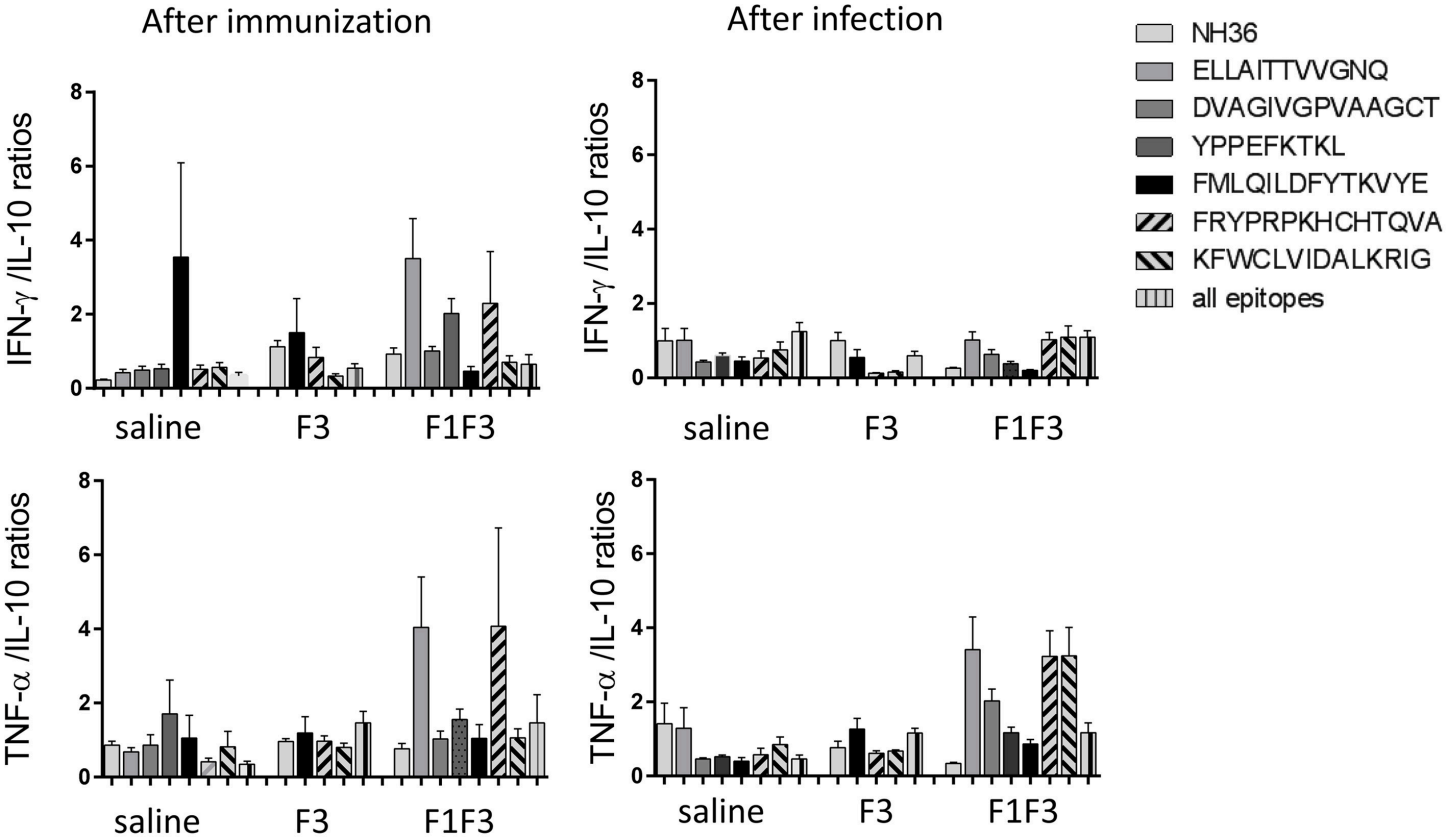
Th1 cytokine responses in supernatants of splenocyte cultures stimulated with T-cell epitopes. Th1 responses are expressed as the calculation of the secreted IFN-γ and TNF-α to IL-10 ratios, after immunization and after challenge. Bars represent the mean ratios + SE of two independent experiments with *n* = 6 animals after immunization, and 7–8 animals after challenge for each treatment.

Moreover, the secretion of cytokines was more pronounced in response to the epitopes than to the NH36 antigen, with the exception of the IFN-γ response to the F3 vaccine after infection (Figure 5). Additionally, we observed that while the ELLAITTVVGNQ, DVAGIVGVPVAAGCT and YPPEKTKL epitopes of F1, and the epitopes FRYPRPKHCCHTQVA and KFWCLVIDALKRIG of the F3 domain are probably correlated to the induction of a Th1 response, the FMLQILDFYTKVYE peptide of F3 induces the secretion of TNF-α and IL-10, to a similar extent, behaving as a probable regulatory T cell epitope. This suggestion was confirmed by calculating the IFN-γ/IL-10 and TNF-α/IL-10 positive ratios (Figure 6). The ELLAITTVVGNQ and the FRYPRPKHCCHTQVA peptides before infection, and both together with the KFWCLVIDALKRIG peptide after infection determined the most potent IFN-γ and TNF-α*/*IL-10 ratios in mice vaccinated with the chimera. In contrast, the FMLQILDFYTKVYE epitope, generated a high IFN-γ*/*IL-10 ratio before infection in the saline-treated controls but promoted the most reduced IFN-γ*/*IL-10 ratios after infection, and TNF-α*/*IL-10 ratios before and after infection, which are indicative of its potential ability to induce a T cell regulatory response (Figure 6).

### The F3 and F1F3 Chimera Induce High Frequency of Multi-Functional CD4+/CD8+ T Cells

The frequencies of NH36 specific CD4^+^ and CD8^+^ T cells secreting IL-2, TNF-α and IFN-γ were assessed alone, or in any combination of two cytokines and three cytokines simultaneously, in order to observe the impact of the vaccines during the advancement of the TH1 CD4^+^ and the CD8^+^ responses.

**Figure 7:**
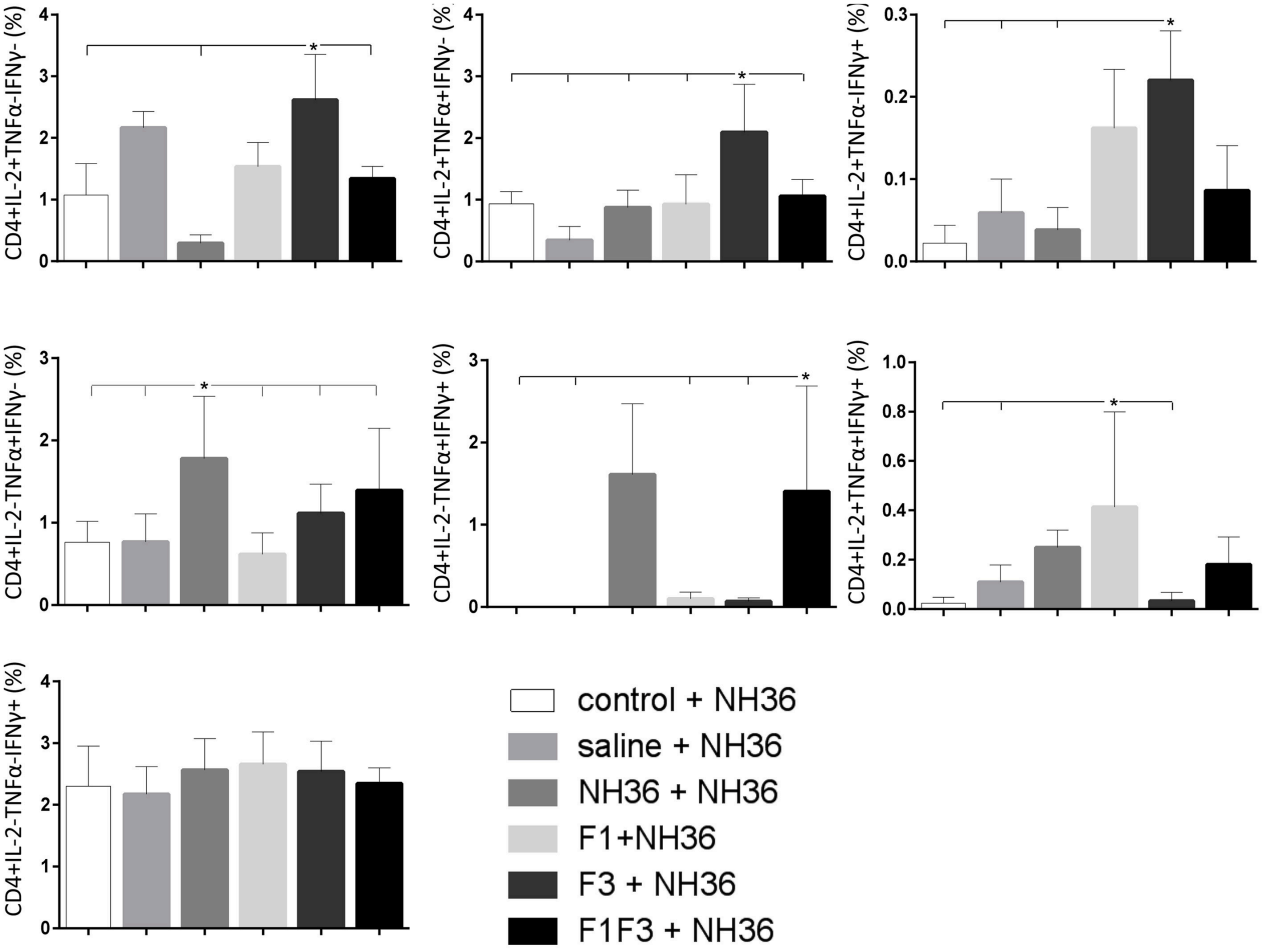
Analysis of intracellular expression of IL-2, IFN-γ and TNF-α, any combination of two cytokines and the three cytokines simultaneously in CD4^+^ T cells stimulated with NH36, after immunization. Splenocyte cultures were stimulated with 25 μg/well of recombinant NH36 for 24 h. Brefeldin 10 mg/ml was added for 4 h. Cells were harvested and labeled with anti-CD4-Fitc, anti-IL-2-PercP, anti-TNF-α-PE, and anti-IFN-γ-APC. Data are means + SE of two independent experiments, each one with 8–10 animals per treatment. Statistical differences were assessed using the Kruskal Wallis and Mann Whitney methods. Asterisks ^*^ and horizontal lines indicate significant differences between treatments (*p* < 0.05).

**Figure 8:**
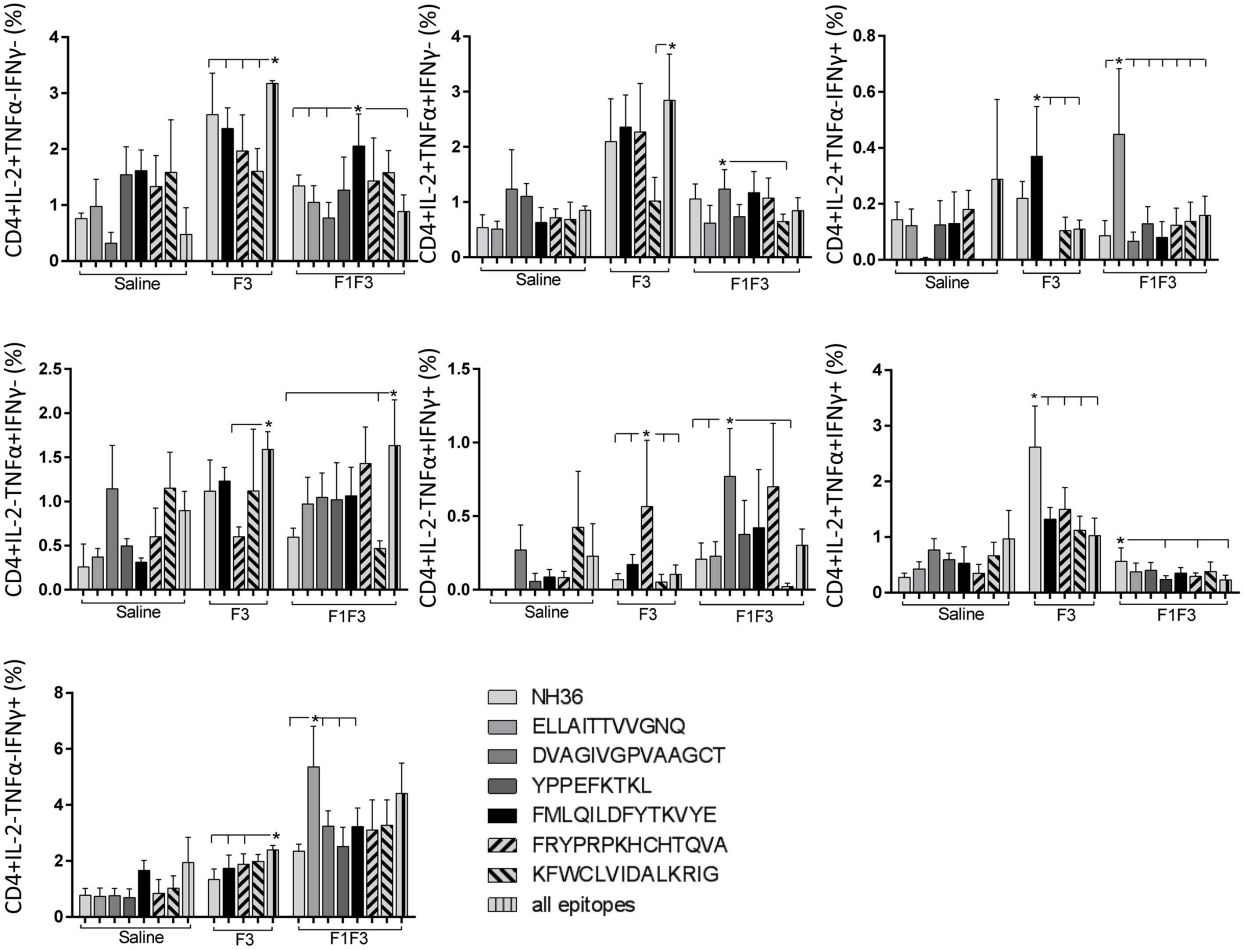
Analysis of intracellular expression of IL-2, IFN-γ, and TNF-α, any combination of two cytokines and the three cytokines simultaneously in CD4^+^ T cells stimulated with epitopes, after immunization. Splenocytes of mice vaccinated with the F3 domain were incubated *in vitro* for 24 h with 25 μg*/*ml of NH36, and with each of the predicted CD4^+^ epitopes for the F3 domain (FMLQILDFYTKVYE, FRYPRPKHCCHTQVA and KFWCLVIDALKRIG) or with the mixture of all the epitopes, after immunization. In addition, splenocytes of the control infected mice and of mice vaccinated with 100 μg of the F1F3 chimera were incubated with each one of the predicted CD4^+^ epitopes for the F1 domain (ELLAITTVVGNQ and DVAGIVGVPVAAGCT) and the F3 domain (FMLQILDFYTKVYE, FRYPRPKHCCHTQVA, and KFWCLVIDALKRIG), and with the highly scored epitope for CD8^+^ T cells of the F1 domain (YPPEKTKL), after immunization and after infection. Brefeldin 10 mg/ml was added for 4 h. Cells were harvested and labeled with anti-CD4-Fitc, anti-IL-2-PercP, anti-TNF-α-PE, and anti-IFN-γ-APC. Data are means + SE of two independent experiments, each one with 8–10 animals per treatment. Statistical differences were assessed using the Kruskal Wallis and Mann Whitney methods. Asterisks ^*^ and horizontal lines indicate significant differences between treatments (*p* < 0.05).

The CD4^+^ response after immunization showed that the F3 vaccine was the most potent and promoted the highest frequencies of single producers of IL-2, and of double producers of IL-2 and TNF-α, and IL-2 and IFN-γ (Figure 7). The NH36 vaccine, in contrast, was predominant for the single producers of TNF-α and, together with the F1F3 chimera, for the double producers of TNF-α and IFN-γ. Furthermore, the F1 vaccine induced the highest frequencies of the multifunctional, triple secretors of IL-2, TNF-α and IFN-γ (Figure 7). In agreement, the F3 vaccine promoted the highest frequencies of most CD4+ cytokine secretor subtypes, in response to all epitopes (Figure 8). The chimera vaccine, in contrast, increased the frequencies of single producers of IFN-γ, and double producers of TNF-α and IFN-γ and of IL-2 and IFN-γ, mainly in response to the ELLAITTVVGNQ peptide, and of single producers of TNF-α and double producers of TNF-α and IFN-γ, in response to the FRYPRPKHVHTQVA peptide (Figure 8). The FMLQILDFYTKVYE epitope, only enhanced the proportions of the single producers of IL-2 (Figure 8).

**Figure 9:**
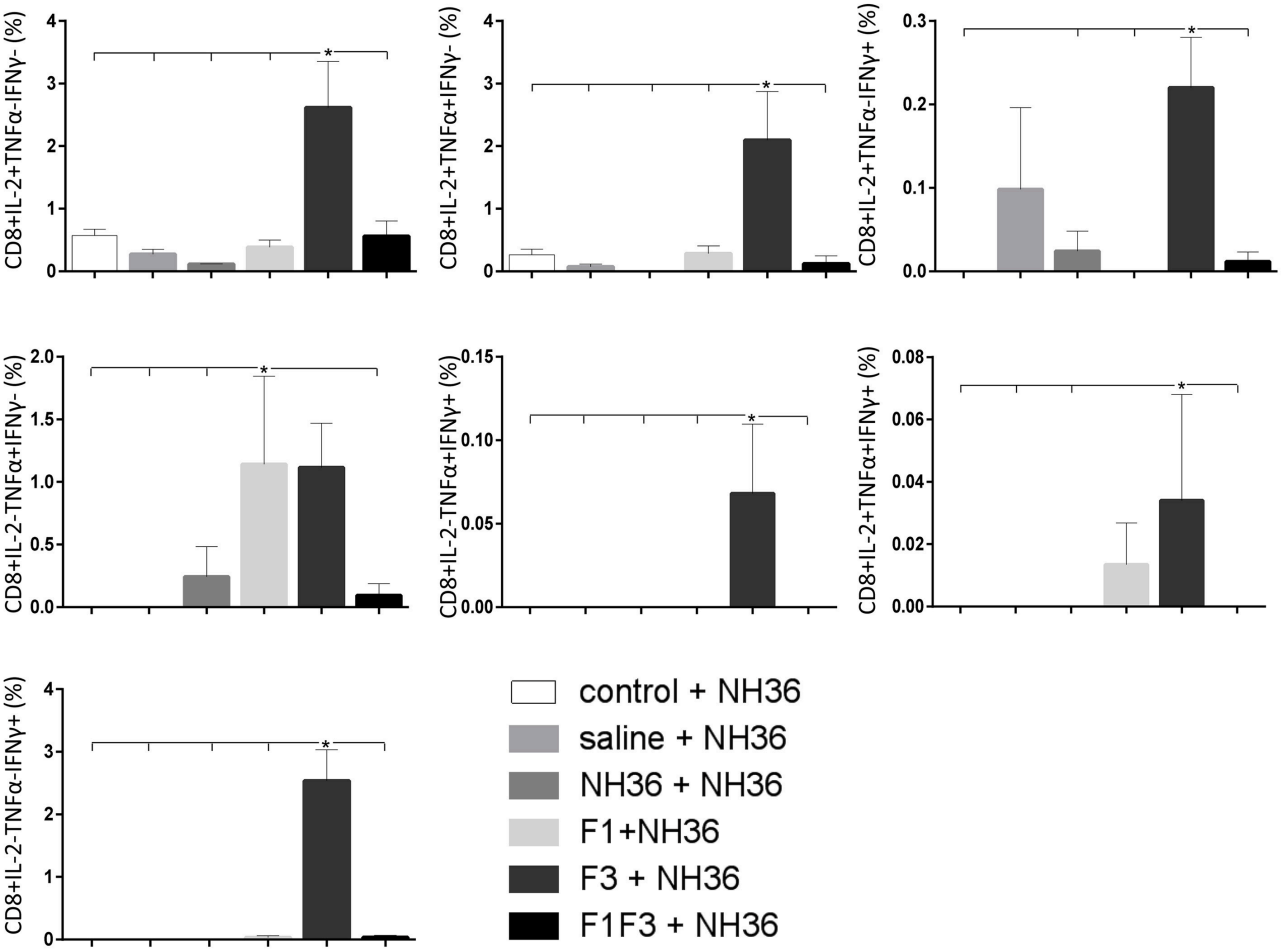
Analysis of intracellular expression of IL-2, IFN-γ and TNF-α, any combination of two cytokines and the three cytokines simultaneously in CD8^+^ T cells stimulated with NH36, after immunization. Splenocyte cultures were stimulated with 25 μg/ml of recombinant NH36 for 24 h. Brefeldin 10 mg/ml was added for 4 h. Cells were harvested and labeled with anti-CD8-Fitc, anti-Il-2-PercP, anti-TNF-α-PE, and anti-IFN-γ-APC. Data are means + SE of two independent experiments, each one with 8–10 animals per treatment. Statistical differences were assessed using the Kruskal Wallis and Mann Whitney methods. Asterisks ^*^ and horizontal lines indicate significant differences between treatments (*p* < 0.05).

**Figure 10:**
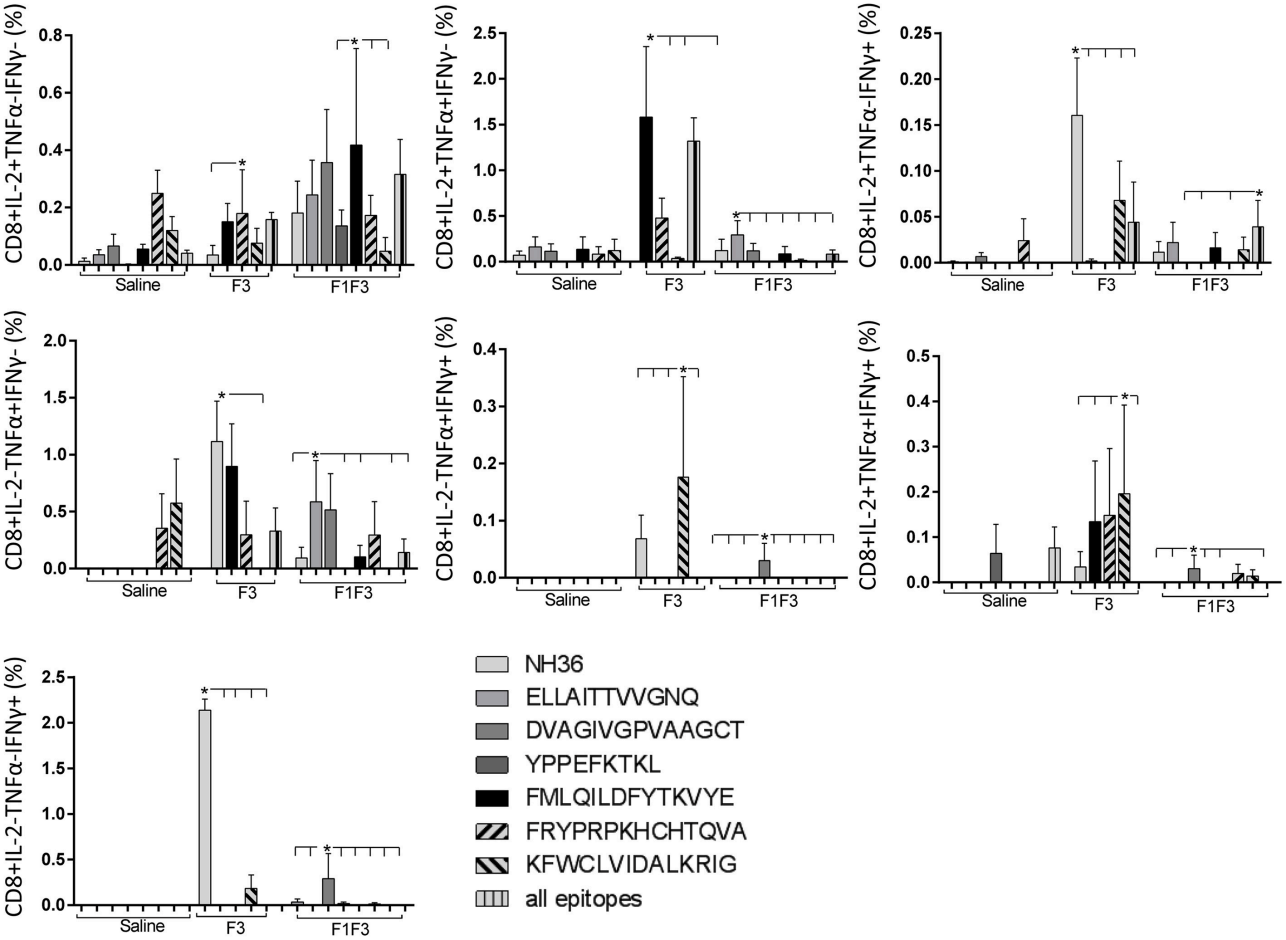
Analysis of intracellular expression of IL-2, IFN-γ and TNF-α, any combination of two cytokines and the three cytokines simultaneously in CD8^+^ T cells stimulated with epitopes, after immunization. Splenocytes of mice vaccinated with the F3 domain were incubated *in vitro* for 24 h with 25 μg*/*ml of NH36, and with each of the predicted CD4^+^ epitopes for the F3 domain (FMLQILDFYTKVYE, FRYPRPKHCCHTQVA, and KFWCLVIDALKRIG) or with the mixture of all the epitopes, after immunization. In addition, splenocytes of the control infected mice and of mice vaccinated with 100 μg of the F1F3 chimera were incubated with each one of the predicted CD4^+^ epitopes for the F1 domain (ELLAITTVVGNQ and DVAGIVGVPVAAGCT) and F3 domain (FMLQILDFYTKVYE, FRYPRPKHCCHTQVA, and KFWCLVIDALKRIG), and with the highly scored epitope for CD8^+^ T cells of the F1 domain (YPPEKTKL), after immunization and after infection. Brefeldin 10 mg/ml was added for 4 h. Cells were harvested and labeled with anti-CD4-Fitc, anti-IL-2-PercP, anti-TNF-α-PE, and anti-IFN-γ-APC. Data are means + SE of two independent experiments, each one with 8–10 animals per treatment. Statistical differences were assessed using the Kruskal Wallis and Mann Whitney methods. Asterisks ^*^ and horizontal lines indicate significant differences between treatments (*p* < 0.05).

The CD8^+^ T cell response after immunization was also predominantly enhanced by the F3 vaccine, which increased the proportions of all the cytokine secretor subtypes, and was as potent as the F1 vaccine, for the single producers of TNF-α and triple producers of IL-2, TNF-α and IFN-γ T (Figure 9). The FMLQILDFYTKVYE epitope was predominant for the frequencies of the single producers of IL-2 of the chimera vaccine, and the double producers of TNF-α and IL-2 of the F3 vaccinated mice, while the KFWCLVIDALKRIG sequence was more potent for the double producers of TNF-α and IFN-γ, and of IL-2 and IFN- γ, and for the triple producers of IL-2, TNF-α and IFN-γ of the F3-vaccinated animals (Figure 10). Additionally, the ELLAITTVVGNQ and DVAGIVGPVAAGCT peptides of F1 also promoted high frequencies of single producers of IL-2 or TNF-α^+^, in mice vaccinated with the chimera (Figure 10).

**Figure 11:**
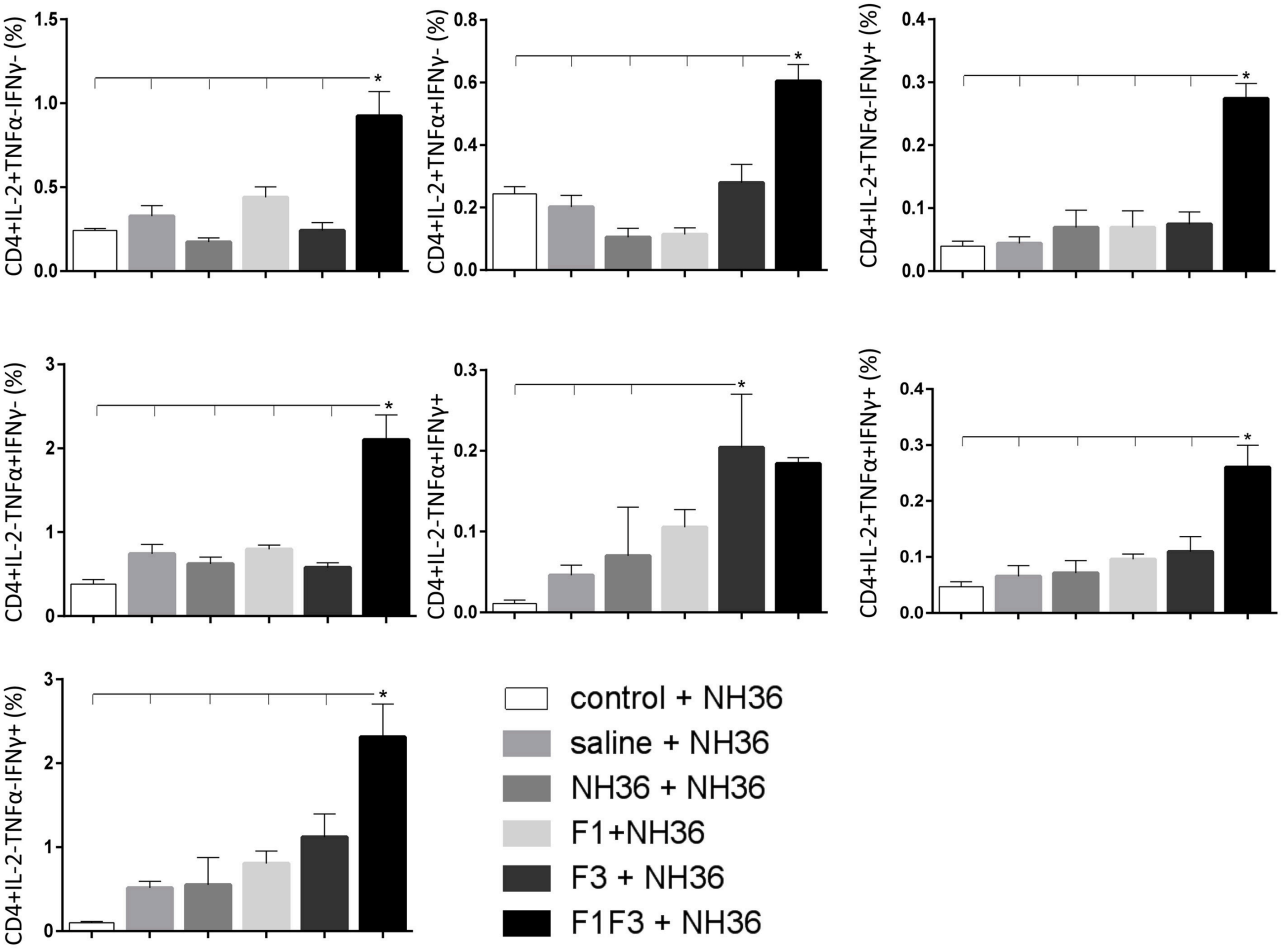
Analysis of intracellular expression of IL-2, IFN-γ and TNF-α, any combination of two cytokines and the three cytokines simultaneously in CD4^+^ T cells stimulated with NH36, after infection. Splenocyte cultures were stimulated with 25 μg/ml of recombinant NH36 for 24 h. Brefeldin 10 mg/ml was added for 4 h. Cells were harvested and labeled with anti-CD4-Fitc, anti-IL-2-PercP, anti-TNF-α-PE, and anti-IFN-γ-APC. Data are means + SE of two independent experiments, each one with 8–10 animals per treatment. Statistical differences were assessed using the Kruskal Wallis and Mann Whitney methods. Asterisks ^*^ and horizontal lines indicate significant differences between treatments (*p* < 0.05).

**Figure 12:**
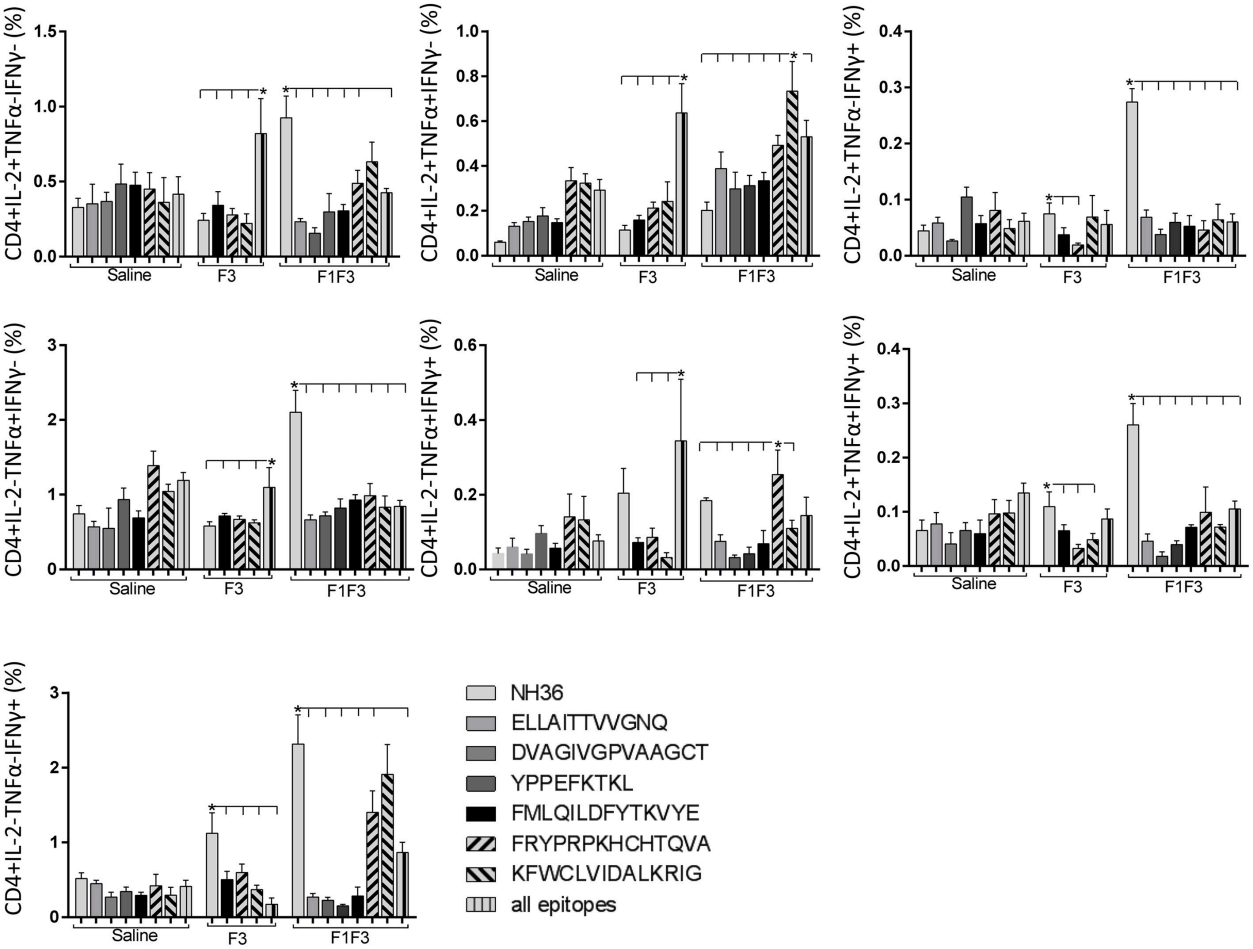
Analysis of intracellular expression of IL-2, IFN-γ and TNF-α, any combination of two cytokines and the three cytokines simultaneously in CD4^+^ T cells stimulated with epitopes, after infection. Splenocytes of mice vaccinated with the F3 domain were incubated *in vitro* for 24 h with 25 μg*/*ml of NH36, and with each of the predicted CD4^+^ epitopes for the F3 domain (FMLQILDFYTKVYE, FRYPRPKHCCHTQVA and KFWCLVIDALKRIG) or with the mixture of all the epitopes, at the end of week 8 after infection. In addition, splenocytes of the control infected mice and of mice vaccinated with 100 μg of the F1F3 chimera were incubated with each one of the predicted CD4^+^ epitopes for the F1 domain (ELLAITTVVGNQ and DVAGIVGVPVAAGCT) and F3 domain (FMLQILDFYTKVYE, FRYPRPKHCCHTQVA, and KFWCLVIDALKRIG), and with the highly scored epitope for CD8^+^ T cells of the F1 domain (YPPEKTKL), after immunization and after infection. Brefeldin 10 mg/ml was added for 4 h. Cells were harvested and labeled with anti-CD4-Fitc, anti-IL-2-PercP, anti-TNF-α-PE, and anti-IFN-γ-APC. Data are means + SE of two independent experiments, each one with 8–10 animals per treatment. Statistical differences were assessed using the Kruskal Wallis and Mann Whitney methods. Asterisks ^*^ and horizontal lines indicate significant differences between treatments (*p* < 0.05).

In contrast to the predominance of the F3 vaccine after vaccination, the main CD4^+^ T cell response after challenge was observed in mice vaccinated with the F1F3 chimera, that showed the highest frequencies of all CD4^+^ cytokine secretor subtypes (Figure 11) mainly directed against the KFWCLVIDALKRIG epitope, which increased the proportions of the single producers of IL-2, IFN-γ and double producers of TNF-α and IL-2, while the FRYPRPKHCCHTQVA peptide enhanced the frequencies of the double producers of TNF-α and IFN-γ (Figure 12).

**Figure 13:**
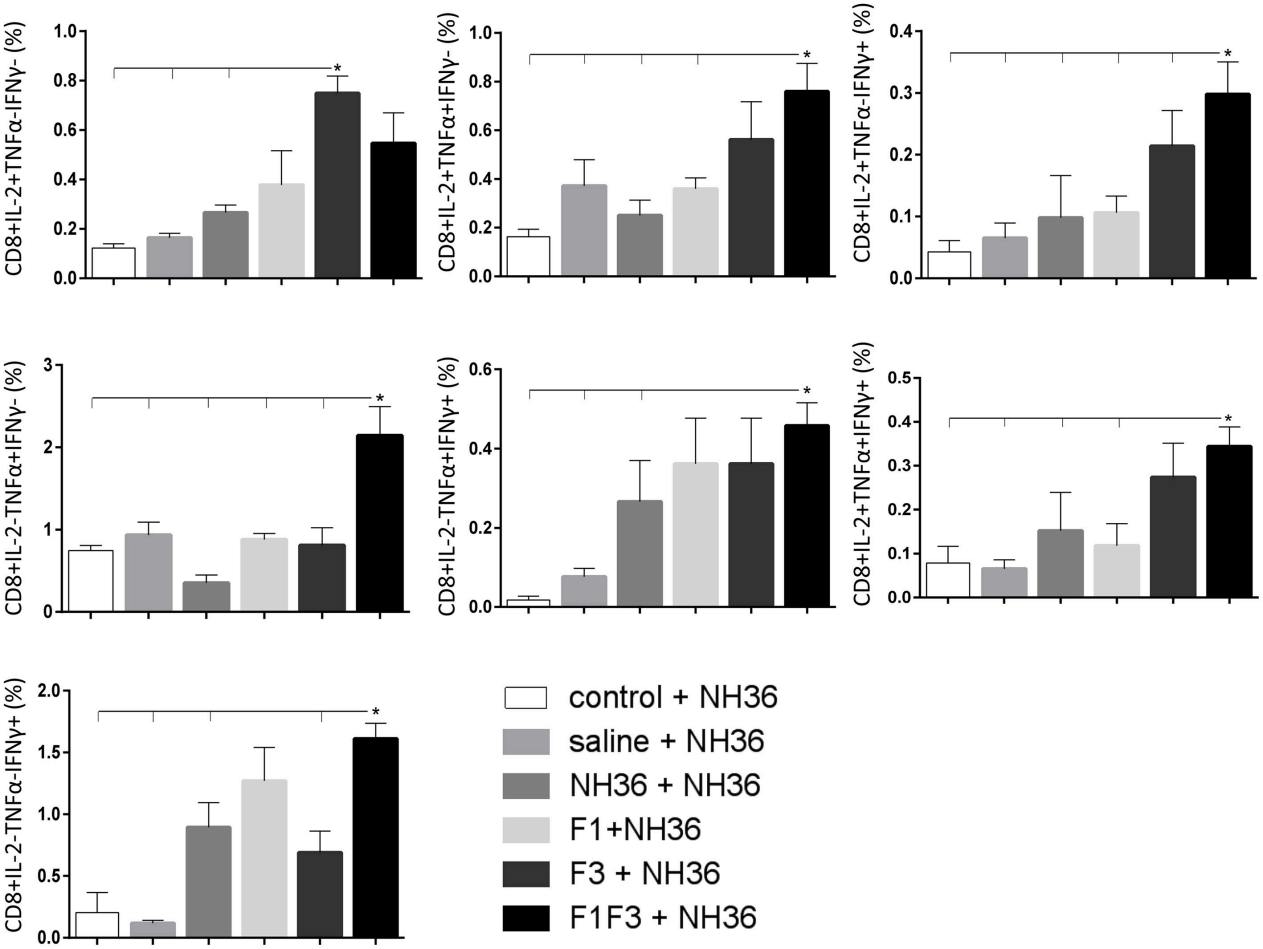
Analysis of intracellular expression of IL-2, IFN-γ and TNF-α, any combination of two cytokines and the three cytokines simultaneously in CD8 T cells stimulated with NH36, after infection. Splenocyte cultures were stimulated with 25 μg/ml of recombinant NH36 for 24 h. Brefeldin 10 mg/ml was added for 4 h. Cells were harvested and labeled with anti-CD8-Fitc, anti-Il-2-PercP, anti-TNF-α-PE, and anti-IFN-γ-APC. Data are means + SE of two independent experiments, each one with 8–10 animals per treatment. Statistical differences were assessed using the Kruskal Wallis and Mann Whitney methods. Asterisks ^*^ and horizontal lines indicate significant differences between treatments (*p* < 0.05).

**Figure 14:**
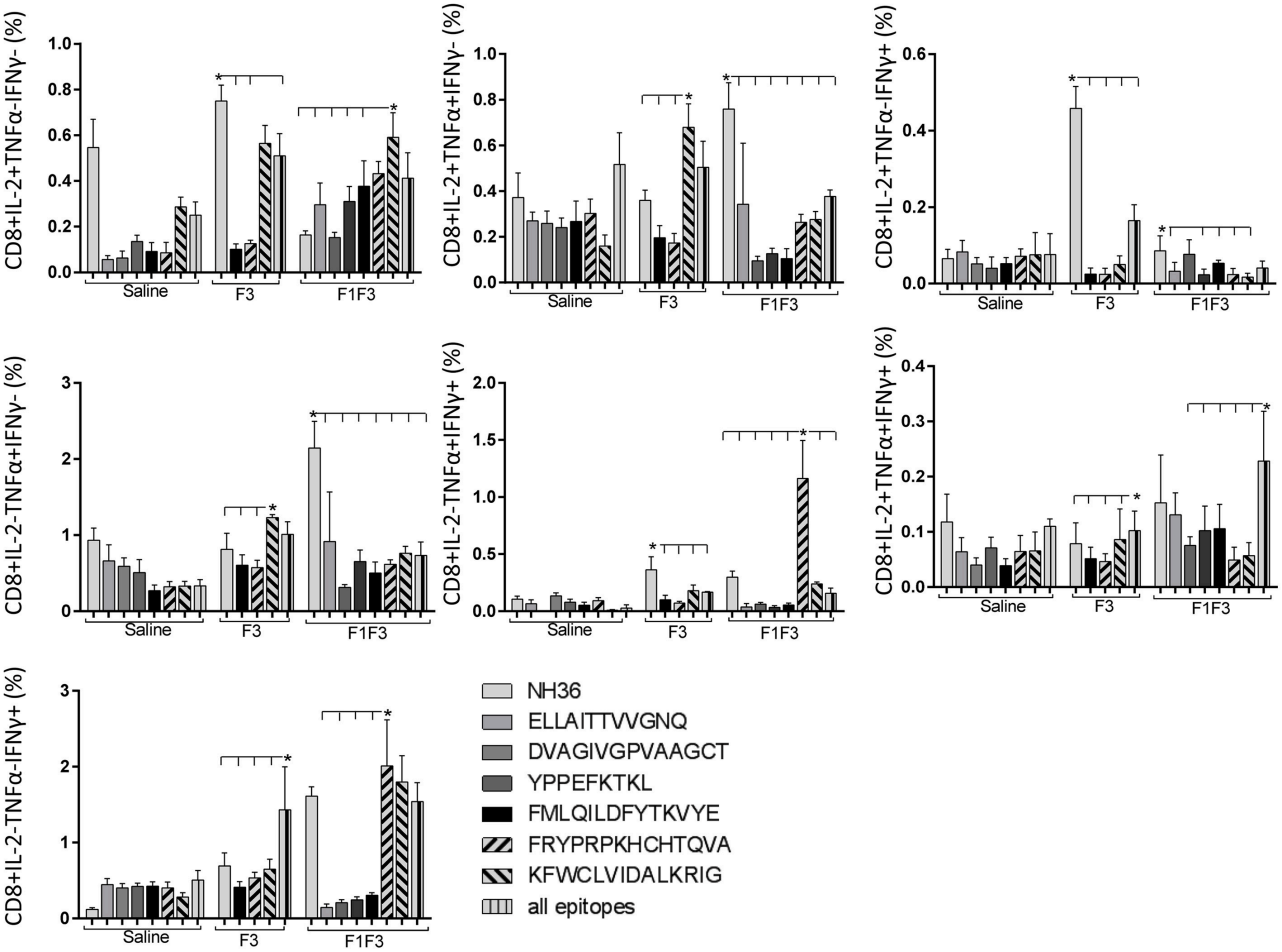
Analysis of intracellular expression of IL-2, IFN-γ and TNF-α, any combination of two cytokines and the three cytokines simultaneously in CD8^+^ T cells stimulated with epitopes, after infection. Splenocytes of mice vaccinated with the F3 domain were incubated *in vitro* for 24 h with 25 μg*/*ml of NH36, and with each of the predicted CD4^+^ epitopes for the F3 domain (FMLQILDFYTKVYE, FRYPRPKHCCHTQVA and KFWCLVIDALKRIG) or with the mixture of all the epitopes, at the end of week 8 after infection. In addition, splenocytes of the control infected mice and of mice vaccinated with 100 μg of the F1F3 chimera were incubated with each one of the predicted CD4^+^ epitopes for the F1 domain (ELLAITTVVGNQ and DVAGIVGVPVAAGCT) and F3 domain (FMLQILDFYTKVYE, FRYPRPKHCCHTQVA, and KFWCLVIDALKRIG), and with the highly scored epitope for CD8^+^ T cells of the F1 domain (YPPEKTKL), after immunization and after infection. Brefeldin 10 mg/ml was added for 4 h. Cells were harvested and labeled with anti-CD4-Fitc, anti-IL-2-PercP, anti-TNF-α-PE and anti-IFN-γ-APC. Data are means + SE of two independent experiments, each one with 8–10 animals per treatment. Statistical differences were assessed using the Kruskal Wallis and Mann Whitney methods. Asterisks ^*^ and horizontal lines indicate significant differences between treatments (*p* < 0.05).

Likewise, the chimera promoted the strongest CD8^+^ T cell response after infection, but shared its predominance with the F3 vaccine, for the frequencies of single producers of IL-2 and double producers of TNF-α and IL-2, and TNF-α and IFN-γ and multifunctional CD8 T cells (Figure 13). The FRYPRPKHCCHTQVA epitope alone enhanced the frequencies of the double producers of TNF-α and IFN-γ (Figure 14), while in combination with KFWCLVIDALKRIG, increased the proportions of the single producers of IL-2 or IFN-γ of mice vaccinated with the chimera.

We concluded that the variation of the frequencies of CD4^+^ or CD8^+^ T cells induced by the vaccines were significant and quite expressive. We compared the four vaccines and observed that the F3 formulation determined a significant enhancement of frequencies of CD4^+^ and CD8^+^ secreting-T cells after immunization (Figures 7, 9), while the chimera vaccine was predominant for both types of T cells after infection (Figures 11, 13). The responses to incubation with the epitopes were sometimes homogeneous indicating that the results of the epitope-prediction programs were correct, and that most of the synthetic epitopes indeed represent the most immunogenic regions of the NH36 sequence. Our results therefore confirm the relevant functions of all tested sequences. Nevertheless, it was possible to detect that the ELLAITTVVGNQ, FRYPRPKHCHTQVA, and FMLQILDFYTKVYE epitopes predominated in the induction of the CD4^+^ response (Figure 8), and the FMLQILDFYTKVYE peptide prevailed in the CD8 response after immunization (Figure 10), while the FRYPRPKHCHTQVA and KFWCLVIDALKRIG sequences dominated the CD4^+^ (Figure 12) and CD8^+^ responses after infection (Figure 14).

### Vaccine Efficacy Is Enhanced by the F1F3 Chimera

**Figure 15:**
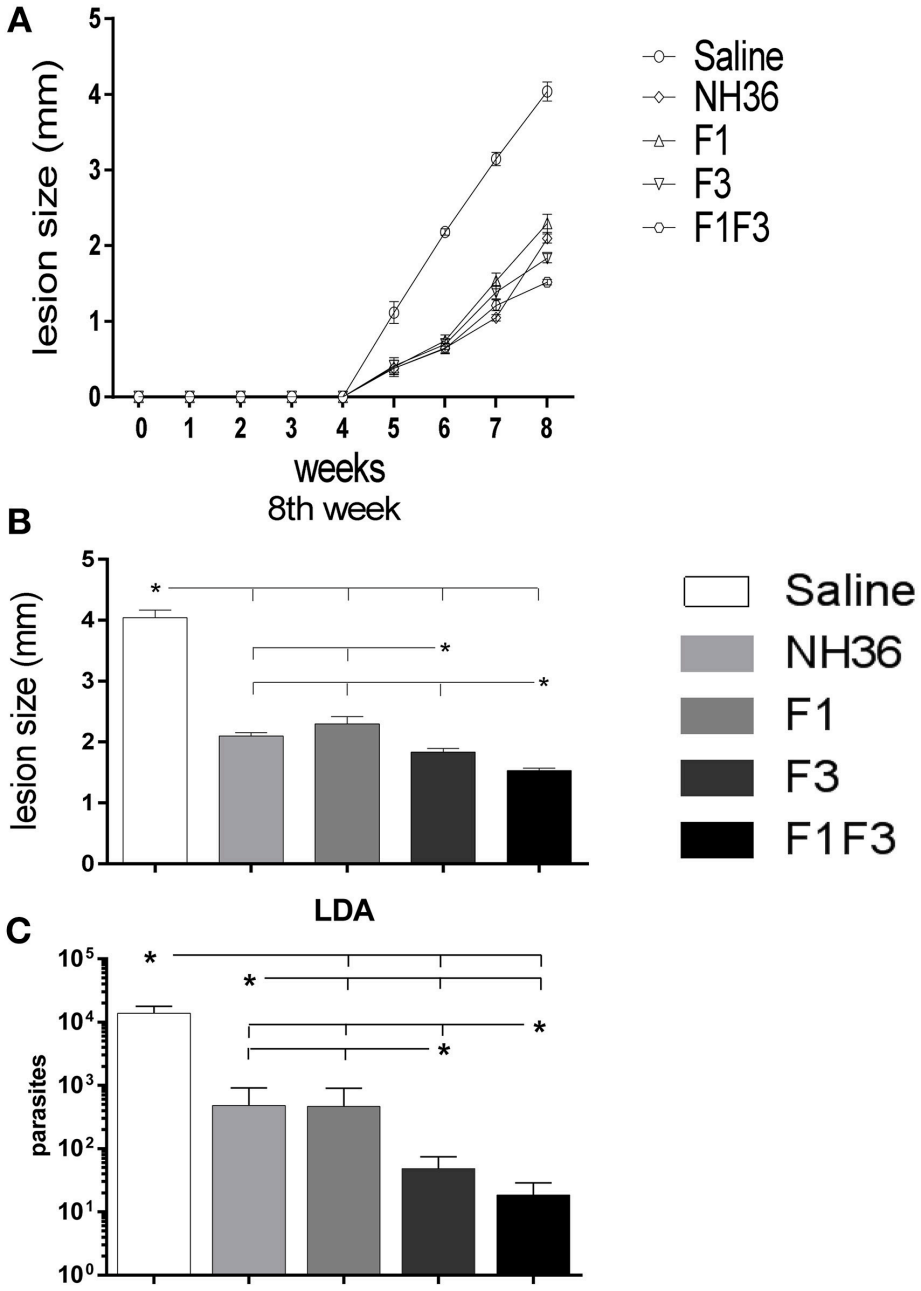
Analysis of vaccine efficacy. **(A)** Sizes of the ear lesions. Curves show the evolution of the sizes of lesions over time (mean + SE) in mm from two independent experiments with *n* = 10 animals per treatment in each experiment. **(B)** Sizes of the ear lesion at the end of week 8 after infection. **(C)** Reduction of the parasitic load on infected ears. The parasite load of the infected ears were evaluated after *in vitro* culture using the limiting dilution technique. Statistical differences were assessed using the Kruskal Wallis and Mann Whitney methods **(A,B)** and the IC95% test was used for the analysis of the parasite load. Data are means + SE of two independent experiments, each one with 8–10 animals per treatment. Asterisks ^*^ and horizontal lines indicate significant differences between treatments.

The evolution of the infection was monitored by the increase in cutaneous lesion sizes of the ears until the end of the eighth week after infection. With the exception of the F1 vaccine, all formulations induced protection and decreased lesion sizes compared to saline controls (Figure 15).

At the end of week 8th, the F1F3 chimera induced the most potent reduction of the lesion sizes when compared to the saline controls (62%). The F3 vaccine reduced the lesions by 55% while the NH36 and F1 vaccines determined a 48 and 43% reduction, respectively. These differences were significant (*p* < 0.05). The assessment of the parasite load in the lesions revealed the same performances, however, these differences between vaccines were not significant.

The measures of the ear lesions during week 8 after infection and the log_10_ number of parasites in lesions were highly correlated (*p* < 0.001, *R* = 0.5048, *R*^2^ = 0.2548).

In agreement, the LDA results were also very illustrative. All vaccines reduced the parasite load significantly in comparison to the infected controls that showed a mean value of 13,784 parasites. While the NH36 and the F1 vaccines promoted similar levels of protection, showing 482 and 468.5 parasites (96 and 96.6% of reduction of parasite load, respectively), the F3 vaccine reduced the parasite burden to 48.5 parasites (99.6% of protection), and the chimera, to 18.5 parasites, what represented the maximal protection (99.8%) (Figure 15C).

Furthermore, the increases in the antibody response after infection were good surrogates for protection. Significant negative correlations were identified between the increases of IgG (*p* < 0.0137, *R* = –0.3168, *R*^2^ = 0.1003), IgG1 (*p* < 0.0005, *R* = –0.4370, *R*^2^ = 0.1910), IgG2a (*p* < 0.0026, *R* = –0.3819, *R*^2^ = 0.1459), IgG2b (*p* < 0.0009, *R* = –0.4189, *R*^2^ = 0.1755) and the IgG3 antibody levels (*p* < 0.0024, *R* = –0.3854, *R*^2^ = 0.1485) and the decrease in the number of parasites in the lesions.

Additionally, after infection, the IDR, the levels of TNF-α secreted into supernatants and the frequencies of CD8^+^IFN-γ^+^- secreting T cells were good correlates of protection. In fact, the number of parasites in lesions was negatively correlated with IDR after immunization, at 24 h (*p* < 0.0050, *R* = –0.3575, *R*^2^ = 0.1278) and 48 h (*p* < 0.0041, *R* = –0.3651, *R*^2^ = 0.1333) and IDR after infection, at 24 h (*p* < 0.0047, *R* = –0.3603, *R*^2^ = 0.1298) and 48 h (*p* < 0.0092, *R* = –0.3336, *R*^2^ = 0.1113). The parasite load in lesions was also negatively correlated to the levels of TNF-α secreted into supernatants (*p* < 0342, *R* = –0.2739, *R*^2^ = 0.0750) and to the frequencies of CD8^+^IFN-γ^+^-secreting T cells (*p* < 0.0500, *R* = –0.3530, *R*^2^ = 0.1246) meaning that protected mice show enhanced TNF-α secretion and higher proportions of CD8^+^ T cells secreting IFN-γ^+^.

Therefore, among all the vaccines tested, the F1F3 and F3 vaccines showed the highest immunogenicity and efficacy (Supplementary Table 1). We concluded that the F1F3 chimera vaccine was superior in the induction of antibodies and IDR, before and after infection, and promoted equivalent responses of cytokine secretion against NH36. The chimera induced responses against YPPEKTKL and FMLQILDFYTKVYE epitopes before infection, and together with the F3 vaccine, against the FRYPRPKHCCHTQVA, KFWCLVIDALKRIG, and FMLQILDFYTKVYE peptides, after challenge. The F3 vaccine induced higher IFN/IL-10 and TNF-IL-10 ratios than the F1F3 chimera, which exhibited an increased TNF-α and IL-10 secretion indicative of a T-regulatory performance, at all times, mainly directed against the FMLQILDFYTKVYE peptide. Generally, the F3 vaccine induced the most potent CD4^+^ Th1 and CD8^+^ T cell responses before infection, while the F1F3 chimera was more efficient after challenge. The FMLQILDFYTKVYE epitope was responsible for the simultaneous secretion of TNF-α and IL-10 in supernatants. The ELLAITTVVGNQ and DVAGIVGPVAAGCT peptides of F1 acted more on cytokine secretion by CD4 and CD8 T cells before infection while the FRYPRPKHCCHTQVA, KFWCLVIDALKRIG did that after challenge. Furthermore, the F1F3 chimera induced the strongest reduction of lesion sizes, but the parasite loads were equivalent in the chimera or the F3-vaccinated mice. Thus, we therefore conclude that both vaccines induced a strong cross-immunity against infection by *L. (V.) braziliensis*. While the F3 vaccine determines an earlier trigger of the development of the CD4^+^ and CD8^+^ T cell responses, the F1F3 was more efficient in long-term infection.

## Discussion

NH36 is a conserved phylogenetic marker of the genus *Leishmania* (42), a vital enzyme for the metabolism of the DNA of the parasite (43) and a very potent antigen (32, 33, 35, 37–39, 44, 45). Accordingly, it is considered a strong candidate for the development of a cross-protective vaccine against both visceral and cutaneous leishmaniasis of mice and humans (35, 38, 39, 41). Having previously identified that the F3 domain of NH36 contains important epitopes involved in the CD4^+^ lymphocyte mediated protection against visceral leishmaniasis caused by *L*. (*L*.) *infantum chagasi* (38) and against cutaneous leishmaniasis produced by *L. (L.) amazonensis* (35) we aimed, in this work, to design of a multi-epitope vaccine against leishmaniasis. The F3 vaccine induced the maximal survival and a long-lasting immune protection with an early CD4^+^ Th1 response, high IFN-γ and TNF-α/IL-10 ratios and frequencies of CD4^+^ T cells secreting IL-2^+^, TNF-α^+^, or IFN-γ^+^, or any combination of the two or the three cytokines simultaneously (IL-2^+^TNF-α^+^IFN-γ^+^) (46). Additionally, we recently demonstrated that the F3-vaccine preserved the maturation, migration properties and CCR7 expression of DCs, which are essential for the generation of cell-mediated immunity (46). On the other hand, the F1 domain of NH36 holds the epitopes involved in the CD8^+^ T cell mediated protective response against cutaneous leishmaniasis caused by *L*. (*L*.) *amazonensis* (35) and promotes the simultaneous secretion of IFN-γ, TNF-α, and IL-10 (35).

We previously demonstrated that vaccination with the F1 and F3 domains cloned in tandem, as a chimera vaccine, optimized protection against cutaneous leishmaniasis by *L*. (*L*.) *amazonensis* above the levels achieved by vaccination with either of the domains injected separately or admixed together (39). This is in agreement with the theory that T cell multi-epitope vaccines have greater efficacy when antigens that contain enriched frequencies of the relevant epitopes are used.

The NH36 amino acid of *L*. (*L*.) *donovani* shows 99% and 93% of identity to the sequences of NHs of *L*. (*L*.) *chagasi* (36) and *L*. (*L*.) *amazonensis* (35, 47), respectively. In contrast, the identity to the NH of *L*. (*V*.) *braziliensis*, which belongs to the *Leishmania* subgenus *Viannia*, is slightly lower (84%) (36, 47). In fact, a few amino acid substitutions were noticed in the sequences of its epitopes restricted to HLA-DR and HLA-A and B haplotypes for humans (41). However, since *L*. (*V*.) *braziliensis* is the most frequent etiological agent of CL and ML in Latin America, and since ML is a very severe and disfiguring syndrome of leishmaniasis, the assay of the cross-protective capabilities of the NH36 vaccine against infection by *L*. (*V*.) *braziliensis* was very important.

In this investigation we first aimed to identify if the F1 and F3 domains of NH36 are immunoprotective against *L*. (*V*.) *braziliensis* infection. Second, we studied if the vaccine antigen presented as a recombinant chimera would be more immunogenic than the use of the domains independently. Finally, we aimed at the identification of the most relevant epitopes to be used in a potential future synthetic multi-epitope vaccine against *L*. (*V*.) *braziliensis* infection.

The comparative analysis of the antibody response in sera demonstrated that the F1F3 chimera was more potent than the NH36, F1, and F3 vaccines and enhanced the NH36 specific IgA and IgG2a response before and after infection. Additionally, the chimera enhanced the IgG and IgG3 levels after challenge. These results are indicative of an enhancement of the Th1 response which has been associated with the induction of IgG2a, IgG2b, and IgG3 antibodies (48). Moreover, IgG2a, IgG2b, and IgG3 together are more capable of fixing complement than IgG1 (49) and that IgG3 can cooperatively bind to microorganisms (50) and provide protection (51). An enhanced IgG1 and IgG2a response against the antigen was obtained by vaccination with polyproteins of *L*. (*V*.) *braziliensis* formulated with saponin. This mixed response was expected for the use of saponin as adjuvant (35, 37, 38) and was observed after the use of the F1F3 chimera against the *L*. (*L*.) *amazonensis* infection in mice (39). The immunogenic potential of the chimera vaccine on the humoral response might be related to the previously described peptides 17 and 18 (52) that overlap with the sequences AVQKRVKEVGTKPAAFML and NQTLEKVTRNARLVADVAG of the F3 and F1 domains (38), also contained in the chimera (39). Interestingly, the peptide 17 showed a 100% of sensitivity in the diagnosis of canine and human VL (52). Thus, our results allow us to speculate about the potential cross-reactivity of these B cell epitopes of NH36 in the context of the infection by *L*. (*V*.) *braziliensis*.

Furthermore, the F1F3 chimera improved the IDR response and promoted a 49% enhancement above the levels induced by NH36, while the F1 vaccine promoted the lowest IDR. A positive reaction to the leishmanial skin test (LST), as well as lymphocyte proliferation and production of high levels of IFN-γ and TNF-α are characteristic of the immune response of CL due to *L*. (*V*.) *braziliensis* infection (10, 53, 54). The Montenegro skin test (MST), with a sensitivity rate of 86.4 up to 100% is the main diagnostic test in primary care (55) and is also positive in asymptomatic subjects indicating previous exposure to *Leishmania* infection but without disease (56, 57). Interestingly, in field assays of a crude vaccine against CL, vaccine efficacy was only detected among vaccinated individuals that showed positive MST to leishmanial antigen (58–60). Remarkably, the NH36-saponin vaccines induced strong IDR responses which were excellent correlates of protection against mice infected by *L*. (*L*.) *infantum chagasi* and *L*. (*L*.) *amazonensis* (35, 37–39). Additionally, the F1F3 chimera generated stronger IDR responses than NH36 against *L*. (*L*.) *amazonensis* infection, above the levels promoted by the F1 or F3 domains (39). As a good correlate of protection, the IDR responses of chimera vaccinated mice challenged with *L*. (*V*.) *braziliensis* were positively correlated to the IFN-γ and TNF-α secretion and to the NH36 specific IgG, IgG2a, IgG2b, and IgG3 antibodies. Thus, the improvement promoted by the F1F3 chimera on the IDR response against infection by *L*. (*V*.) *braziliensis* indicates that this vaccine might be used for prophylaxis against all forms of cutaneous leishmaniasis. Supporting our findings, Carvalho et al. (5) reported that in human studies, the IDR test, rather than IFN-γ production, represents a better estimation of lasting immunity to leishmaniasis and a better tool for the detection of vaccine induced immunity.

After immunization, the secretions of IFN-γ, TNF-α, and IL-10 in response to the NH36 antigen were enhanced more by the F1F3 chimera than by all the other vaccines. The NH36 vaccine also enhanced the TNF-α secretion whereas, after infection, the F3 vaccine induced the strongest IFN-γ and TNF-α secretions while the chimera was dominant for production of IL-10. The levels of the three cytokines were correlated with increases of IDR and IgG2a and IgG3 antibodies indicating a mixed and/or T-cell regulatory response. Confirming that, strong Th1 responses were disclosed by the elevated IFN-γ*/*IL-10 and TNF-α*/*IL-10 ratios promoted by the F3 vaccine, before infection and detected in the F1 vaccinated and infected controls after challenge with *L*. (*V*.) *braziliensis*. In contrast, the F1F3 chimera reduced the pathological Th1 response followed by the NH36 vaccines, which is an extremely desired effect for a vaccine against mucosal leishmaniasis.

The Th1 response and IFN-γ are strongly exacerbated in mucosal leishmaniasis due to *L*. (*V*.) *braziliensis* infection (10). In fact, while IFN-γ secretion is correlated to protection or natural resistance against visceral leishmaniasis (41, 61) an excessive IFN-γ response is associated to the pathology of CL and ML caused by *L*. (*V*.) *braziliensis* (5, 62). Although the IFN-γ secretion by CD4^+^ and CD8^+^ T cells was related to a protective response against *L*. (*V*)*. braziliensis* infection, cells from patients infected with *L*. (*V*)*. braziliensis* secrete higher amounts of IFN-γ (63). The excessive IFN-γ and TNF-α secretion is a signal of a lack of regulation of the pro-inflammatory response and in fact, the high frequencies of cells expressing IFN-γ were associated to low frequencies of cells expressing the IL-10 receptor (16) or IL-10 (6, 17, 18) after infection with *L*. (*V*.) *braziliensis*. Bacellar et al. (10) considered that the high production of IFN-γ and TNF-α in patients, concomitant to a decreased capability of IL-10 and TGF-β to modulate this effect are the abnormalities that justify the pathological characteristics of the disease.

In this scenario, our findings gain more relevance since, while a high Th1 responses with high IFN-γ*/*IL-10 and TNF-α*/*IL-10 ratios were observed in the *L*. (*V*.) *braziliensis* infected controls, in contrast, the F1F3 chimera vaccine showed strong regulatory capabilities linked to the high secretion of IL-10 that downmodulated the inflammatory response. Our findings support our previous description of an enhanced IFN-γ, TNF-α and IL-10 secretion after vaccination with F1 in mice further challenged with *L*. (*L*.) *amazonensis* infection (35, 37, 38). This mixed response was enriched and stronger when we used the F1F3 chimera, which also contains the F1 domain and enhanced the IFN-γ, TNF-α, and IL-10 secretions as well (39).

We tried to identify which NH36 epitope is responsible for the induction of the Th1 and of the T regulatory response against the *L*. (*V*.) *braziliensis* infection.

While the ELLAITTVVGNQ, DVAGIVGVPVAAGCT, YPPEKTKL, FRYPRPKHCCHTQVA, and KFWCLVIDALKRIG epitopes are probably correlated to the induction of a Th1 response only, the FMLQILDFYTKVYE peptide stimulates the secretion of IFN-γ, TNF-α and IL-10 and therefore is probably also responsible for the T cell regulatory response. These indications should be confirmed by the analysis of the presence of markers of T cell regulatory response in splenocytes incubated with the FMLQILDFYTKVYE epitope. We previously detected these mixed and T cell regulatory capabilities of the YPPEKTKL and FMLQILDFYTKVYE epitopes in mice vaccinated with the F1F3 chimera and challenged with *L*. (*L*.) *amazonensis* (39). Our results therefore confirm that they can be used in a cross-protective vaccine and in a universal T cell epitope vaccine against leishmaniasis. Interestingly, Tregs have been described as the principal sources of IL-10 in these patients infected with *L*. (*V*.) *braziliensis* (64).

After immunization, the F3 vaccine, together with the NH36 and F1 vaccines stimulated the secretion of IL-2, TNF-α and IFN-γ cytokines by CD4^+^ T cells. This Th1 response was mainly directed against the ELLAITTVVGNQ, FRYPRPKHCCHTQVA KFWCLVIDALKRIG, and FMLQILDFYTKVYE epitopes while the CD8^+^T cell response was directed against the FMLQILDFYTKVYE and KFWCLVIDALKRIG sequences.

In contrast, after challenge, the F1F3 chimera promoted the most prominent CD4^+^ and CD8^+^ cytokine-secreting T cell responses, which were directed against the KFWCLVIDALKRIG and FRYPRPKHCCHTQVA epitopes of F3. Only after challenge was it possible to detect the predominant frequencies of the multifunctional CD4^+^ and CD8^+^IL-2^+^TNF-α^+^IFN-γ^+^ T cells that were promoted by the chimera vaccine, and which are correlated to a memory immune response (40). In CL, the CD8^+^ T cells are involved both in the pathology and in the protection against the disease (13). Depletion of CD8+ T cells in BALB/c mice infected with *L*. (*V*.) *braziliensis* resulted in reduced lesion sizes (64). Although no depletion of CD8^+^ T cells was performed in this investigation, the correlation between the decrease in the sizes of parasite lesions and the increase in frequencies of CD8^+^IFN-γ^+^ secreting T cells in mice vaccinated with the chimera indicates the CD8^+^ T cell involvement in the chimera vaccine induced-protection.

Similar to what we described previously in BALB/c immunized with the F1F3 chimera vaccine but challenged with *L*. (*L*.) *amazonensis*, the FMLQILDFYTKVYE epitope promoted a regulatory response and the KFWCLVIDALKRIG, a Th1 response (39).

Finally, the evolution of the lesion sizes revealed that the F1F3 chimera was the most potent vaccine to reduce the sizes of ear lesions, followed by the F3, F1, and NH36 in decreasing order. A very similar heitghtened performance of the F1F3 chimera was observed against the cutaneous infection caused by *L*. (*L*.) *amazonensis* in mice (39). In addition, the analysis of the parasite load by LDA was useful to disclose those variables that were good correlates of protection. In fact, the number of parasites decreased in negative correlation with the increases of IgG, IgG1, IgG2a, IgG2b, and IgG3 antibody levels, IDR, the levels of TNF-α secreted into supernatants and the frequencies of CD8^+^IFN-γ^+^- secreting T cells. In addition, LDA was able to disclose significant differences between the performance of the vaccines, and in agreement with the measure of the size of lesions, revealed the chimera as the most potent vaccine, which was followed in efficacy by the F3 vaccine. In contrast, the NH36 and F1 vaccines induced lower protection.

In our investigation, we aimed to reproduce the model of the ear infection of BALB/c mice with cutaneous leishmaniasis caused by *L. (L.) major* (40, 65, 66) but using a *L. (V.) braziliensis* challenge instead. In previous investigations LDA has been extensively used with success for parasite load determinations (39, 65–68). These are all investigations dealing with cutaneous lesions, where the parasite can not be counted microscopically in stained smears. Darrah et al. (65), Belkaid et al. (66), Castillo et al. (67), Duarte et al. (68), Alves-Silva et al. (39) and a large number of papers used Limiting dilution assays for the quantification of the parasites in cutaneous lesions. Although a quantitative PCR technique is also used for parasite load determination, the LDA methodology proved to be adequate. Confirming our assumption, Castillo et al. (67) considered the LDA method as the golden standard and described that the real-time PCR assay for *Leishmania* subgenus showed a very good linear correlation with quantification on the basis of the limiting dilution assay (*R*^2^ = 0.975–0.938) in experimentally infected mice. Confirming that in the present investigation the number of parasites detected in lesions by LDA was negatively correlated to the IDR measures, after immunization and after infection, and to the levels of TNF-α secreted to supernatants and the frequencies of CD8^+^IFN-γ^+^-secreting T cells, giving a very good indication of the achieved vaccine efficacies.

One limitation of our study is the fact the Balb/c model does not reproduce the lesions of mucosal leishmaniasis of humans. The hamster model of *L. (V.) braziliensis* infection can reproduce cutaneous lesions similar to those observed in humans, with no healing (69). Unfortunately, the wider use of hamsters is limited due to the lack of antibodies for cell markers and cytokine (70). The Macaca mulata rhesus monkey model, on the other hand, induces self-healing CL with parasite resolution and lesional granulomas similar to those developed by humans (71). This model can also be used to elucidate the regulatory mechanisms (72), however, is not suitable for large vaccination experiments. The Balb/c model of dermal infection of the ear with 10^5^ parasites, that we used in our investigation, does not reproduce the Mucosal leishmaniasis of humans, but closely resembles the American Cutaneous leishmaniasis caused by *L (V.) braziliensis*, since it shows the development of ulcerated lesions that heal spontaneously, parasite dissemination to lymphoid organs and development of a Th1 response (73).

Our results indicate that the F3, more than the F1 domain of NH36 independently, contributes to the generation of protection against *L*. (*V*.) *braziliensis*. However, the presentation of both domains in tandem as the F1F3 chimera increases protection, probably by modulating the exacerbated pro-inflammatory response through its regulatory capability. In addition, we identified the most potent immunogenic epitopes responsible for the Th1 pro-inflammatory and the T-cell regulatory responses. These epitopes and recombinant chimera of NH36 might possibly be used in a multi-species cross-protective vaccine against cutaneous leishmaniasis and a future multiepitope universal vaccine against leishmaniasis.

## Ethics Statement

This study was performed following the guidelines of the National Institute of Health (NIH, USA). The protocol was approved by the Ethics committee for the use of animals in scientific experimentation (CEUA) of the Centro de Ciências da Saúde of the Universidade Federal do Rio de Janeiro (CONCEA) under the number 01200.001568/2013-87, protocol n°054/17. The animals used in this investigation were raised at the Biotério Central of the Centro de Ciências da Saúde da UFRJ and kept during the experiments, in the facilities of Instituto de Microbiologia Paulo de Góes. They were given water and food *ad libitum* and were maintained at 22°C with 12 h light/dark cycles. In all experiments we aimed to reduce any animal suffering to a minimum.

## Author Contributions

MA-S and DN conducted the experiments. MA-S and DN acquired the data. MA-S and CP analyzed the data. CP and DN designed the research studies. PdL provided reagents. MA-S and CP wrote the manuscript. All authors have read and approved the final manuscript.

### Conflict of Interest Statement

DN and CP are inventors of the patent file PI1015788-3 (INPI Brazil). The remaining authors declare that the research was conducted in the absence of any commercial or financial relationships that could be construed as a potential conflict of interest.

## Supplementary Material

Supplementary Table 1 Superiority of the F3 or F1F3 vaccines for each test variable.

Supplementary Figure 1 Sequence analysis of Nucleoside hydrolases of *Leishmania donovani* (NH36) and *Leishmania (V.) braziliensis*. The sequences of the Nucleoside hydrolases NH36 of *L. (L.) donovani* (Ld-NH36) and XP001564081 of *Leishmania (V.) braziliensis* (Lb-NH) were aligned using the BLASTP Genbank program. The line in the middle of the two sequences shows the amino acids share by the two NHs. The peptide sequence of MHC class II-IA^d^ and IE^d^, haplotype H^2^ CD4+ T cell epitopes (38) are shown in black squares, on the F1 and F3 fragments. The amino acid sequence of the MHC class I L^d^-CD8+ T cell predicted epitope of the F1 fragment (38) is underlined in the gray square.
