# Supplementary material for: The F1F3 Recombinant Chimera of Leishmania donovani-Nucleoside Hydrolase (NH36) and Its Epitopes Induce Cross-Protection Against Leishmania (V.) braziliensis Infection in Mice
Source: Front Immunol. 2019 Apr 9;10:724. doi: 10.3389/fimmu.2019.00724 (PMC6465647; doi:10.3389/fimmu.2019.00724)
Supplement: Supplementary Table 1 — Superiority of the F3 or F1F3 vaccines for each test variable. [file Table_1.pdf]

Supplementary Table 1. Superiority of the F3 or the F1F3 chimera vaccines in each tested

Variable	After immunization	After infection
Anti –NH36 antibodies	F1F3	F1F3
IDR	F1F3	F1F3
Secreted cytokines	F1F3<br>YPPEFKTKL for IFN-γ e TNF-α and FMLQILDFYTKVIE for TNF-α and IL-10	F3 and F1F3<br>FRYPRPKHCHTQVA, KFWCLVIDALKRIG, FMLQILDFYTKVIE for TNF-α and IL-10
IFN-γ/IL10 ratio	F3 > F1F3	F3 > F1F3
TFN-α/IL-10 ratio	F3 > F1F3	F3 > F1F3
$CD4^+$ cytokine-secreting T cells in response to NH36	F3 and F1. F1F3 only for TNF-αIFN-γ	F1F3
$CD4^+$ cytokine-secreting T cells in response to epitopes	F3 and F1F3:<br>FMLQILDFYTKVIE<br>ELLAITTVVGNQ<br>FRYPRPKHCHTQVA,<br>KFWCLVIDALKRIG	F1F3:<br>FRYPRPKHCHTQVA,<br>KFWCLVIDALKRIG
$CD8^+$ cytokine-secreting T cells in response to NH36	F3	F1F3
$CD8^+$ cytokine-secreting T cells in response to epitopes	F3:<br>FMLQILDFYTKVIE<br>KFWCLVIDALKRIG<br>F1F3:<br>FMLQILDFYTKVIE<br>KFWCLVIDALKRIG<br>ELLAITTVVGNQ<br>DVAGIVGPVAAGCT	F3:<br>FRYPRPKHCHTQVA<br>F1F3:<br>FRYPRPKHCHTQVA,<br>KFWCLVIDALKRIG
Lesion sizes		F1F3 is stronger than F3
Parasite load in lesions		Not significantly different
