# Supplementary figures and images for: The F1F3 Recombinant Chimera of Leishmania donovani-Nucleoside Hydrolase (NH36) and Its Epitopes Induce Cross-Protection Against Leishmania (V.) braziliensis Infection in Mice
Source: Front Immunol. 2019 Apr 9;10:724. doi: 10.3389/fimmu.2019.00724 (PMC6465647; doi:10.3389/fimmu.2019.00724)

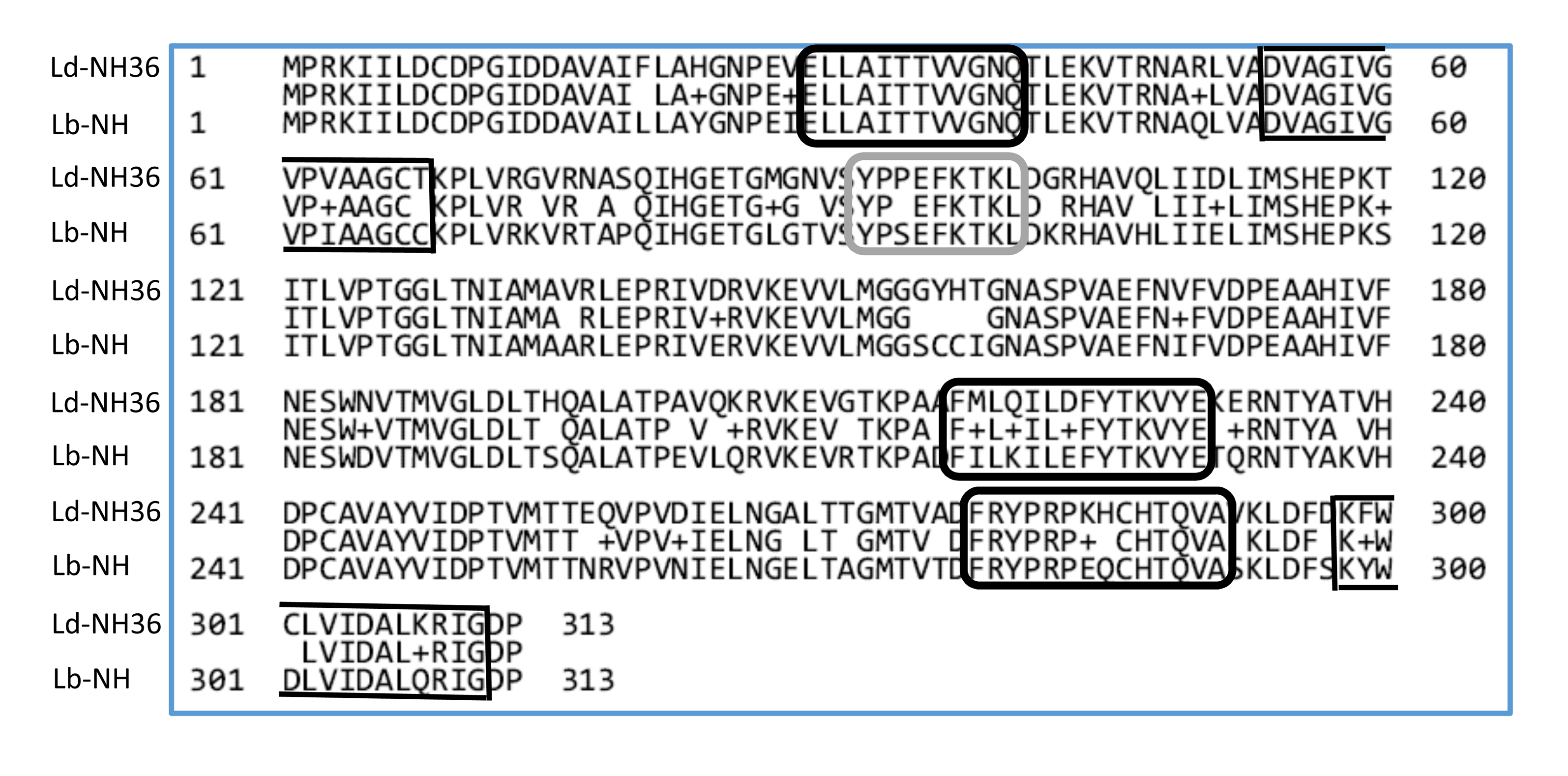

Supplement: Supplementary Figure 1 — Sequence analysis of Nucleoside hydrolases of Leishmania donovani (NH36) and Leishmania (V.) braziliensis. The sequences of the Nucleoside hydrolases NH36 of L. (L.) donovani (Ld-NH36) and XP001564081 of Leishmania (V.) braziliensis (Lb-NH) were aligned using the BLASTP Genbank program. The line in the middle of the two sequences shows the amino acids share by the two NHs. The peptide sequence of MHC class II-IAd and IEd, haplotype H2 CD4+ T cell epitopes (38) are shown in black squares, on the F1 and F3 fragments. The amino acid sequence of the MHC class I Ld-CD8+ T cell predicted epitope of the F1 fragment (38) is underlined in the gray square. [file Image_1.TIF]
